# ETTAS: a modular aptamer-recruited platform for programmable translational activation

**DOI:** 10.1093/nar/gkag692

**Published:** 2026-07-25

**Authors:** Aolin Li, Xiaoting Zhang, Senmao Li, Yifan Wang, Chaojie Xu, Changning Lv, Min Zhao, Yuchen Liu, Mingxia Ding, Congcong Cao

**Affiliations:** Department of Urology, The First Affiliated Hospital of Shenzhen University, Shenzhen Second People’s Hospital, Shenzhen 518035, China; Central Laboratory, Shenzhen Bao’an District Songgang People’s Hospital, Shenzhen 518035, China; Department of Urology, The Second Affiliated Hospital of Kunming Medical University, Kunming 650101, China; Department of Gastroenterology, Wuhan No. 1 Hospital, Wuhan Hospital of Traditional Chinese and Western Medicine, Wuhan 430065, China; Department of Urology, Peking University First Hospital, Beijing 100034, China; Department of Urology, Peking University First Hospital, Beijing 100034, China; School of Basic Medical Sciences, Hubei University of Chinese Medicine, Hubei Shizhen Laboratory, Wuhan 430065, China; Shenzhen Institute of Translational Medicine, Shenzhen Second People’s Hospital, The First Affiliated Hospital of Shenzhen University, Health Science Center, Shenzhen University, Shenzhen 518035, China; Department of Urology, The First People’s Hospital of Yunnan Province, The Affiliated Hospital of Kunming University of Science and Technology, Kunming 650032, China; School of Basic Medical Sciences, Hubei University of Chinese Medicine, Hubei Shizhen Laboratory, Wuhan 430065, China; Shenzhen Institute of Translational Medicine, Shenzhen Second People’s Hospital, The First Affiliated Hospital of Shenzhen University, Health Science Center, Shenzhen University, Shenzhen 518035, China

## Abstract

Precise enhancement of endogenous protein synthesis offers a reversible therapeutic strategy without permanent genomic modification. However, existing Cas13-mediated translational activation systems are limited by modest potency and restricted modular expandability. Here, we developed the Enhanced Targeted Translational Activation System (ETTAS), a modular RNA-guided platform that combines dCas13a, the SINEB2 translational activation element, and an independently recruitable aptamer-mediated auxiliary module. Systematic ortholog screening identified dCas13a as the most effective scaffold for SINEB2-mediated translational activation, whereas direct tandem duplication of SINEB2 elements impaired rather than enhanced activity. To overcome this architectural limitation, we used aptamer-mediated recruitment to spatially separate target recognition from auxiliary activation. A binding-validated, non-interfering dCas13a-binding aptamer enabled construction of a dual-module system in which an aptamer-recruited SINEB2 element enhanced translation without altering target mRNA abundance or stability. Compared with the previously reported dCasRx–SINEB2 system, ETTAS produced stronger reporter activation, stronger endogenous induction of P53 and PTEN, and greater antiproliferative and pro-apoptotic effects in bladder cancer cells. Proteomic analyses showed selective target protein upregulation with limited global perturbation. *In vivo*, dual-AAV delivery of ETTAS activated endogenous P53 and suppressed tumor growth. ETTAS establishes a programmable framework for modular post-transcriptional upregulation of endogenous proteins.

## Graphical Abstract

**Graphical Abstract:**
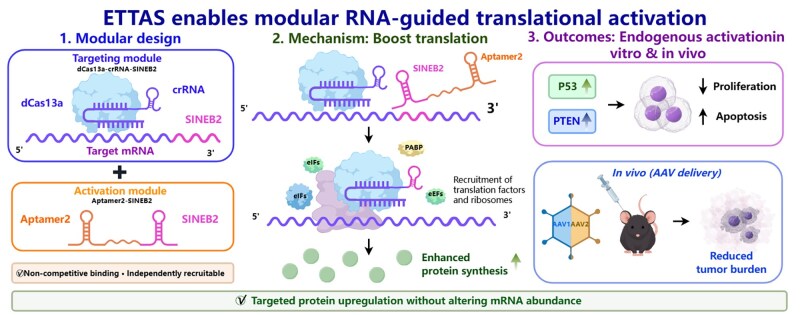

## Introduction

Precise regulation of gene expression at the translational level offers a method to modulate protein output without permanently altering genomic sequences or substantially perturbing endogenous transcriptional programs [1, 2]. Compared with transcriptional regulation, translational control enables rapid, reversible, and highly dynamic modulation of protein synthesis, making it particularly attractive for synthetic biology applications and therapeutic intervention in complex diseases such as cancer [1, 2]. Increasing evidence suggests that dysregulated protein synthesis contributes substantially to tumor progression, therapeutic resistance, and aberrant cellular signaling, which creates demand for programmable technologies capable of selectively enhancing protein production in a target-specific manner [3].

Among emerging RNA-targeting technologies, the CRISPR-associated Cas13 family has gained attention because of its ability to recognize RNA transcripts through programmable guide RNAs [4, 5]. Cas13 orthologs, including Cas13a, Cas13b, Cas13c, and Cas13d (CasRx), have been used for RNA cleavage, RNA editing, RNA imaging, and transcript manipulation in living cells [4–9]. Recently, catalytically inactive Cas13 (dCas13) proteins have been repurposed as programmable RNA-binding scaffolds for post-transcriptional regulation, providing a flexible framework for engineering RNA-guided translational control systems [10, 11].

One promising strategy for translational activation involves incorporation of the SINEB2 element derived from the long non-coding RNA (lncRNA) Uchl1 [12, 13]. SINEB2-containing RNAs have been shown to enhance translation initiation by recruiting translation-associated factors such as eIF4A1 and ILF3, thereby promoting ribosome loading onto target transcripts without substantially altering messenger RNA (mRNA) abundance [14, 15]. Based on this principle, our previous work established a dCasRx–SINEB2 system capable of selectively increasing protein synthesis from endogenous transcripts through RNA-guided targeting, demonstrating the feasibility of programmable translational activation using dCas13-based architectures [16].

Despite these advances, existing Cas13-mediated translational activation systems are limited by weak activation potency and limited modularity [16]. In particular, translational enhancement achieved by a single SINEB2 module is often insufficient for robust protein induction in mammalian cells. Moreover, direct tandem insertion of multiple SINEB2 elements does not necessarily improve translational activation and may instead impair system performance, suggesting the existence of structural and mechanistic constraints that limit simple scaling of translational activation modules. This result suggested that the limitation was architectural rather than simply quantitative, thereby motivating a recruitment-based strategy instead of continued tandem expansion within the CRISPR RNA (crRNA) scaffold. Furthermore, previous systems relied primarily on embedding translational activation elements directly within the targeting crRNA scaffold, thereby coupling target recognition and activation architecture within a single RNA component. These limitations indicate the need for alternative strategies that can enhance translational output while preserving target specificity and enabling modular programmability.

To address these challenges, we explored an aptamer-mediated recruitment strategy for modular translational activation. Rather than directly increasing the copy number of SINEB2 elements within the crRNA scaffold, we sought to recruit auxiliary translational activation modules to the dCas13 complex through engineered RNA–protein interactions [11, 17]. Using SELEX-based screening, we identified multiple Cas13a-binding aptamers and subsequently selected a non-interfering candidate that remained compatible with crRNA loading and target recognition. This strategy established a decoupled modular architecture in which auxiliary translational activation modules could be independently recruited without directly altering the crRNA scaffold itself.

Based on this concept, we engineered the Enhanced Targeted Translational Activation System (ETTAS), a dual-module RNA-guided translational activation platform. ETTAS integrates two cooperative activation modules: a crRNA-linked SINEB2 element that mediates target recognition and primary translational activation, and an independently expressed aptamer-recruited auxiliary SINEB2 module tethered to dCas13a through non-competitive RNA aptamer binding. This modular design substantially enhances translational activation while maintaining transcript specificity and post-transcriptional regulation.

Using reporter systems and endogenous targets, we demonstrate that ETTAS increases protein expression without significantly affecting target mRNA abundance or stability. Compared with previously reported dCasRx–SINEB2 systems, ETTAS consistently achieves stronger translational activation and greater downstream biological effects. Furthermore, ETTAS efficiently activates endogenous tumor suppressor proteins including P53 and PTEN in bladder cancer cells, resulting in inhibition of tumor cell proliferation and induction of apoptosis.

Finally, we demonstrate that ETTAS can be implemented *in vivo* using a dual-AAV delivery architecture for systemic administration. In bladder cancer xenograft models, AAV-mediated ETTAS delivery achieved efficient translational activation of endogenous P53 and suppressed tumor growth. These findings establish ETTAS as a modular and programmable framework for RNA-guided translational engineering and provide a foundation for developing next-generation therapeutic strategies based on programmable post-transcriptional control of endogenous protein synthesis.

## Materials and methods

### Cell lines and cell culture

Human embryonic kidney (HEK293T) cells and bladder cancer cell lines (T24 and 5637) were cultured in Dulbecco’s Modified Eagle Medium (DMEM) supplemented with 10% fetal bovine serum (FBS) and 1% penicillin–streptomycin at 37°C with 5% CO_2_. HEK293T cells were used to evaluate protein synthesis activation systems, while T24 and 5637 cells were used to test ETTAS-mediated activation of tumor suppressor genes P53 and PTEN. Cells were seeded at 1–2 × 10^5^ cells per well in 24-well or 6-well plates and transfected at 60%–80% confluency using Lipofectamine 3000 (Thermo Fisher Scientific) according to the manufacturer’s protocol. Transfection efficiency was monitored using GFP or Fluc reporters and typically ranged from 50% to 70% positive cells. Analyses of protein expression, cell proliferation, and apoptosis were performed 48 h post-transfection.

### Construct design and synthesis

The detailed construction method for dCas13–sgRNA–SINEB2 can be referenced from our previously published article [16]. Vectors encoding dCas13a, dCas13b, and dCas13d, along with their respective crRNAs, were synthesized and cloned into appropriate expression vectors (Supplementary Tables S1–S3 and Supplementary Fig. S1).

We utilized catalytically inactive orthologs of Cas13: dLwaCas13a (Leptotrichia wadei), dPspCas13b (Prevotella sp. P5-125), and dRfxCas13d (Ruminococcus flavefaciens). Coding sequences were either obtained from Addgene or synthesized based on published data. These variants were expressed under the EF1α promoter and fused with a nuclear export signal (NES) to promote cytoplasmic localization.

The SINEB2 element and its derivatives (2× and 3× repeats) were incorporated into the 3′ end of crRNAs using standard cloning techniques to evaluate their impact on translation activation. DNA aptamers for dCas13a were developed through the SELEX (Systematic Evolution of Ligands by Exponential Enrichment) technique, followed by high-throughput sequencing to to identify enriched candidate binders. RNA-form aptamers were then generated from selected DNA candidates for independent functional validation.

Constructs combining SINEB2 elements with RNA aptamers were assembled using overlap extension polymerase chain reaction (PCR) to enhance their translational activation capability. The final constructs, including ETTAS, were sub-cloned into relevant expression vectors for subsequent transfection and *in vitro* and *in vivo* validation of protein expression efficiency. The crRNA sequences were listed in the Supplementary Table S2.

### SELEX technique for aptamer selection

To identify DNA aptamers that specifically bind to the dCas13a protein, an optimized SELEX strategy was employed. A single-stranded DNA library with a 40-nucleotide central random region, flanked by fixed primer-binding sequences, was incubated with immobilized dCas13a protein on magnetic beads. After incubation, unbound sequences were thoroughly washed away, and the remaining DNA–protein complexes were eluted and amplified by PCR. This enriched DNA pool was then used as the input for subsequent SELEX rounds to enhance specificity and affinity.

### SPR analysis of DNA aptamers

Following six rounds of SELEX enrichment and high-throughput sequencing, selected DNA aptamer candidates were subjected to surface plasmon resonance (SPR) analysis using a Biacore T200 instrument to evaluate their binding affinity for dCas13a. DNA aptamers were characterized by SPR because their chemical stability and reduced conformational lability make them well suited for initial label-free kinetic measurements. Equilibrium dissociation constants (*K*_D_) were determined for the best-performing candidates, which were then selected for subsequent functional evaluation and RNA-form derivative testing. SPR was used primarily for initial screening and ranking of DNA aptamer candidates, whereas validation of the RNA-form aptamer relied mainly on BLI and cellular assays.

The top 5 most frequently bound aptamers were selected for the following experiments. Their respective sequences were as follows: Aptamer 1 (CACGCATAACCTGGCAGTAGGTTGCAAACGTCCTCGGTTTCCGCTTGTGTTATGCGTG), Aptamer 2 (CACGCATAACCCACCCCATCTGTCCCGTCCCCCTGCTGTGTCCCTCGTGTTATGCGTG), Aptamer 3 (CACGCATAACCGCGAAGACACCCTTGGCTCTCAGCTCCGTCGCTAGGTGTTATGCGTG), Aptamer 4 (CACGCATAACATGACGTCCTCGGCACTCAGCCCGTCACCGGTAATTGTGTTATGCGTG), Aptamer 5 (CACGCATAACGTCTCAACGGTGACGTCCCCGGCATTCAACTCGCCTGTGTTATGCGTG).

### Molecular docking analysis

To investigate the interaction between dCas13a and the selected aptamers, a molecular docking approach was employed. The three-dimensional (3D) structure of dCas13a was predicted using I-TASSER [18], which provided a protein model suitable for molecular recognition analysis. The 3D structures of both DNA and RNA aptamers were generated using the Vfold3D program based on their predicted secondary structures. Predicted secondary structures of the selected aptamers were first obtained and used as input constraints for Vfold3D-based structural modeling. The resulting models were then used for subsequent docking analysis with dCas13a.

Molecular docking between dCas13a and the aptamers was performed using HDOCK [19], a platform designed for protein-nucleic acid interactions. The docking process employed the ITScoreRP scoring function to evaluate candidate binding configurations. The top-ranked docking models with high-confidence scores were further analyzed to identify putative interaction regions. Visualization and analysis of the predicted protein–aptamer complexes were carried out using PyMOL, focusing on binding domains, spatial relationships, and potential interaction sites. These structural models were used to generate mechanistic hypotheses and guide interpretation of the functional data, rather than to define experimentally validated binding conformations.

### Expression of dCas13a and crRNA-based constructs

For all *in vitro* experiments, dCas13a was expressed from a mammalian expression plasmid under the control of the EF1α promoter. In the ETTAS system, crRNA expression was driven by a U6 promoter, whereas the RNA aptamer was expressed under a CMV promoter, as indicated for each construct. dCas13a and the corresponding RNA-based components were delivered as separate plasmids to allow modular assembly of the system. The use of distinct Pol III and Pol II promoters allows precise expression of crRNA for Cas13a loading while enabling flexible engineering of the aptamer transcript without compromising functional interaction. Non-targeting guide RNA (NT-gRNA) constructs were used as matched negative controls under otherwise identical experimental conditions.

### Guide RNA design

Guide RNAs (gRNAs) were designed to target regions within the coding sequence (CDS) downstream of the start codon to minimize potential interference with translation initiation [20]. During guide selection, local RNA secondary structure and target accessibility were explicitly evaluated using *in silico* RNA folding predictions. Regions predicted to form highly stable hairpins, extensive double-stranded structures, or extreme GC-rich sequences were avoided to reduce steric hindrance and maximize Cas13a binding efficiency. Candidate guides were further assessed empirically in dual-luciferase or GFP reporter assays to confirm functional translation activation, and the most effective guides were selected for downstream experiments. These design criteria ensure that the dCas13a–crRNA complex can efficiently access the target site while allowing elongating ribosomes to displace or bypass the bound protein, enabling robust translational activation without disrupting normal mRNA processing or stability.

### Transfection procedures

HEK293T cells and bladder cancer cell lines (T24 and 5637) were seeded at 1–2 × 10^5^ cells per well in 24-well plates (for reporter or flow cytometry assays) or in 6-well plates (for protein expression analysis) one day prior to transfection to achieve ∼60%–80% confluency. Transfections were performed using Lipofectamine 3000 (Invitrogen) following the manufacturer’s protocol. For HEK293T cells, 1 μg of plasmid DNA per well was used. For bladder cancer cell lines, optimized transfection conditions were applied to ensure consistent delivery of dCas13a and crRNA-based constructs, including ETTAS and corresponding control plasmids. Cells were incubated for 24–48 h post-transfection before subsequent analyses, including protein expression assays, dual-luciferase reporter assays, flow cytometry, and phenotypic evaluations. Quantitative experiments were performed using three independent biological replicates, each containing two technical replicates, and data are presented as mean ± SD.

### Fluorescence microscopy and flow cytometry

Forty-eight hours post-transfection, HEK293T cells were observed under a fluorescence microscope to qualitatively assess GFP expression as an initial indicator of translation activation. For quantitative analysis, cells were harvested, washed twice with cold phosphate-buffered saline (PBS), and resuspended in PBS. Flow cytometry was performed using a BD FACSCanto II, collecting 10 000–20 000 events per sample. During analysis, cellular debris was excluded based on forward and side scatter (FSC/SSC), and doublets were removed using singlet gating (FSC-A versus FSC-H). GFP-positive cells were identified using untransfected cells as negative controls to set fluorescence thresholds.

### Luciferase reporter assays

Dual-luciferase reporter assays were employed to quantify translation activation efficiency in transfected cells. Forty-eight hours post-transfection, HEK293T cells were lysed using Passive Lysis Buffer (Promega), and luciferase activity was measured using the Dual-Luciferase Reporter Assay System (Promega). Firefly luciferase (Fluc) served as the primary reporter, while Renilla luciferase (Rluc) was used as an internal reference to normalize transfection efficiency. This approach enabled the comparison of translation activation efficiencies across different dCas13-based systems and validated the superior performance of the ETTAS in enhancing protein expression.

### Western blot analysis

Cells were lysed using RIPA buffer, and protein concentrations were quantified using the bicinchoninic acid (BCA) protein assay to ensure equal loading. Cell pellets were resuspended in sample buffer, quickly sonicated, boiled, and loaded onto polyacrylamide gels for SDS–PAGE. Proteins were then transferred onto polyvinylidene fluoride (PVDF) membranes for immunoblotting. The following antibodies were used for detection: anti-GFP rabbit polyclonal antibodies (Life Technologies) at a 1:1000 dilution, anti-P53 (Abcam) at 1:1000, anti-GAPDH (Invitrogen) at 1:5000, and anti-PTEN (Invitrogen) at 1:500. After incubation with primary antibodies, membranes were treated with horseradish peroxidase (HRP)-conjugated secondary antibodies (anti-mouse-HRP or anti-rabbit-HRP; Dako) for detection. The ECL detection system (GE Healthcare) was used in conjunction with the Alliance LD2-77WL system (Uvitec, Cambridge) for image detection and analysis.

### Proteomics analysis

For unbiased assessment of global protein expression changes, label-free quantitative proteomics was performed to compare cells expressing the enhanced green fluorescent protein (EGFP)-targeting ETTAS construct with an otherwise identical NT-gRNA control. Cells were harvested at the same time point as EGFP protein analysis, lysed in denaturing buffer containing protease inhibitors, and equal amounts of total protein were subjected to reduction, alkylation, and trypsin digestion following standard bottom-up proteomics procedures. Peptides were desalted and analyzed by nanoLC-MS/MS on a high-resolution mass spectrometer operated in data-dependent acquisition mode.

Raw data were processed using a standard label-free quantification workflow and searched against the UniProt human reference proteome supplemented with the EGFP sequence. Protein identifications were filtered at a 1% false discovery rate at both peptide and protein levels. Protein abundances were quantified based on log2-transformed label-free intensities. Differential expression analysis was performed by comparing EGFP-targeting ETTAS samples with NT-gRNA controls using a two-sided Student’s *t*-test, followed by Benjamini–Hochberg correction for multiple testing. Proteins were considered differentially expressed using combined thresholds of adjusted *P*-value < 0.05 and an absolute log2 fold change ≥ 1.5.

### RNA extraction and RT-qPCR

Total RNA was extracted from plasmid-transfected cells using TRIzol reagent (Invitrogen) according to the manufacturer’s instructions. Extracted RNA was reverse-transcribed into cDNA using the RevertAid First Strand cDNA Synthesis Kit (Thermo Fisher Scientific). Quantitative real-time PCR (RT-qPCR) was performed on an ABI PRISM 7000 system (Applied Biosystems) using All-in-One™ qPCR Mix (GeneCopoeia). The PCR conditions consisted of an initial denaturation at 95°C for 15 min, followed by 40 cycles of 94°C for 15 s, 55°C for 30 s, and 72°C for 30 s.

For endogenous gene expression analyses, GAPDH was used as the reference gene. For luciferase reporter assays, Fluc mRNA levels were normalized to Rluc mRNA levels in the corresponding samples. Relative expression levels were calculated using the 2^−ΔΔCt^ method. All measurements were performed using three independent biological replicates, with two technical replicates per biological replicate, and data are presented as mean ± SD. The primer sequences for GAPDH, Fluc, RLuc, GFP, SINEB2, PTEN, and P53 are presented below, with 5′-to-3′ lengths:

GAPDH-F: TCCCATCACCATCTTCCA
GAPDH-R: CATCACGCCACAGTTTCC
Fluc-F: TACACCTTCGTGACTGCTTC
Fluc-R: CTTGATCTTGGCCTTCAGGA
RLuc-F: GCAACATGGAGTCCGAAATG
RLuc-R: TTGACCTCGGACACCTTTAG
GFP-F: ACGACGGCAACTACAAGACC
GFP-R: TTGTACTCCAGCTTGTGCCC
SINEB2-F: CAGTGCTAGAGGAGGTCAGAAGA
SINEB2-R: GGAGCTAAAGAGATGGCTCAGCACTT
P53-F: CCTCAGCATCTTATCCGAGTGG
P53-R: TGGATGGTGGTACAGTCAGAGC
PTEN-F: TCCCAGACATGACAGCCATC
PTEN-R: TGCTTTGAATCCAAAAACCTTACT

### Bio-layer interferometry assays

Bio-layer interferometry (BLI) assays were performed using an Octet system (FortéBio) to assess the interaction between RNA aptamers and dCas13a. Streptavidin (SA) biosensors were hydrated in running buffer [PBS supplemented with 0.01% Tween-20 and 0.1 mg/ml bovine serum albumin (BSA)] and loaded with 5′-biotinylated RNA ligands, including Aptamer2 WT, Aptamer2 Mut, Aptamer1, or scrambled RNA controls, to a loading response of approximately 0.8–1.0 nm. After establishing a baseline, sensors were exposed to increasing concentrations of purified apo dCas13a (0–800 nM) for 300 s to monitor association, followed by dissociation in running buffer for 360 s.

For crRNA competition experiments, dCas13a was pre-incubated with synthetic crRNA at a molar ratio of 1:1.2 for 30 min at room temperature prior to BLI analysis, and binding responses were compared with those obtained using apo dCas13a under identical conditions. Cold-competitor assays were performed by pre-incubating dCas13a with unlabeled competitor RNAs (10× – 100× molar excess of Aptamer2 WT or scrambled RNA) for 15 min before association with immobilized RNA. All sensorgrams were reference-subtracted using blank sensors, and data were analyzed using Octet Data Analysis software. Each experiment was independently repeated at least three times with consistent results.

BLI was used for RNA aptamers because it provides a flexible and less surface-constrained format for monitoring RNA–protein interactions, which is advantageous for RNA molecules with greater conformational dynamics and potential sensitivity to immobilization conditions.

### Cell viability assay

The cell viability of bladder cancer cells (T24 and 5637) was assessed using the Cell Counting Kit-8 (CCK-8) assay. Cells were seeded into 96-well plates at a density of 5 × 10^3^ cells per well and transfected with constructs of the ETTAS targeting tumor suppressor genes P53 or PTEN. Following transfection, CCK-8 reagent was added to each well, and the cells were incubated for an additional 2 h. Absorbance was measured at 450 nm using a microplate reader to determine cell viability. This assay enabled the evaluation of the inhibitory effects of ETTAS-mediated protein activation on the proliferation of bladder cancer cells.

### Cell apoptosis assay

To evaluate apoptosis induced by ETTAS, T24, and 5637 bladder cancer cells were transfected with the indicated constructs targeting P53 or PTEN. At 48 h after transfection, both floating and attached cells were collected, washed twice with cold PBS, and resuspended in 1× binding buffer. Cells were then stained with Annexin V-FITC and propidium iodide (PI) using an Annexin V-FITC/PI Apoptosis Detection Kit according to the manufacturer’s instructions, followed by incubation in the dark for 15 min at room temperature.

Samples were analyzed on a BD FACSCanto II flow cytometer, and data were processed using FlowJo software. During analysis, cellular debris was first excluded based on forward scatter (FSC) and side scatter (SSC), and doublets were excluded by standard singlet gating. Cells were then classified into four populations based on Annexin V-FITC and PI signals: viable cells (Annexin V−/PI−), early apoptotic cells (Annexin V+/PI−), late apoptotic or secondary necrotic cells (Annexin V+/PI+), and necrotic cells (Annexin V−/PI+). The total apoptotic fraction was calculated as the sum of early apoptotic and late apoptotic cells. All apoptosis assays were performed with three independent biological replicates.

### Immunohistochemistry

Tumor tissues collected from xenograft-bearing mice were fixed in 4% paraformaldehyde overnight at 4°C, embedded in paraffin, and sectioned at a thickness of 5 μm. Sections were deparaffinized in xylene and rehydrated through a graded ethanol series. Antigen retrieval was performed in citrate buffer (pH 6.0) using a pressure cooker. Endogenous peroxidase activity was blocked with 3% hydrogen peroxide for 10 minutes, followed by blocking in 5% BSA for 30 min at room temperature.

Slides were incubated overnight at 4°C with a primary antibody against P53 (1:200 dilution, Abcam ab32389) and then with an HRP-conjugated secondary antibody for 1 h at room temperature. Color was developed using DAB (3,3′-diaminobenzidine) substrate, and sections were counterstained with hematoxylin. Stained sections were visualized using a bright-field microscope, and representative images were captured from multiple tumor regions.

### *In vivo* delivery and tumor growth inhibition

To evaluate the therapeutic potential of ETTAS in inhibiting tumor growth, a bladder cancer xenograft model was established. Female BALB/c nude mice (6–8 weeks old) were subcutaneously injected with 5 × 10^6^ T24 bladder cancer cells into the right flank. When tumors reached approximately 100 mm³, mice were randomly assigned to the indicated treatment groups (*n* = 5 per group).

Due to AAV packaging limitations, a dual-AAV9 delivery strategy was employed. AAV1 encoded the hEF1α promoter-driven dLwaCas13a cassette with a polyA signal. AAV2 contained two expression cassettes: a U6 promoter-driven crRNA fused to a SINEB2 element and a CMV promoter-driven Aptamer2-SINEB2 cassette. A non-targeting (NT) control was included in which the guide RNA sequence did not target the intended transcript, while the same dual-vector delivery framework was preserved.

Each mouse received 5 × 10^11^ vector genomes (vg) of AAV1 and 5 × 10^11^ vg of AAV2 (total 1 × 10^12^ vg/mouse) via tail vein injection. Tumor sizes were measured every 3–4 days using digital calipers, and tumor volumes were calculated as *V* = ½ × *L* × *W*^2^. After 15 days of treatment, mice were euthanized, and tumors were excised and weighed.

Tumor tissues were further subjected to hematoxylin and eosin (H&E) staining and immunohistochemical (IHC) analysis of P53 expression to assess treatment-associated histopathological changes and target protein activation *in vivo*. Absolute *in vivo* stoichiometry of the crRNA–SINEB2 and Aptamer2-SINEB2 RNA components was not directly quantified in the current study.

All animal procedures were approved by the Ethical Committee for Animal Experiments at Shenzhen Second People’s Hospital and performed in accordance with institutional and national guidelines for animal welfare.

### Bladder cancer lung metastasis model and evaluation of anticancer effects

To investigate the therapeutic efficacy of ETTAS in a metastatic setting, a lung metastasis model was established by injecting luciferase-labeled T24 bladder cancer cells (1 × 10^6^ cells/mouse) into the tail vein of female BALB/c nude mice. Seven days post-injection, animals (*n* = 5 per group) were randomly assigned to receive systemic treatment with AAV-ETTAS-P53 or the corresponding non-targeting control AAV-ETTAS-NT. The dual-vector design included AAV1 carrying hEF1α-dLwaCas13a and AAV2 carrying U6-crRNA-SINEB2 and CMV-Aptamer2-SINEB2, each administered at a dose of 1 × 10^11^ viral genomes via tail vein injection on days 7, 10, and 13. In the non-targeting control group, the same dual-vector delivery framework was preserved, while the guide RNA sequence did not target the intended transcript.

Tumor progression was assessed weekly by bioluminescence imaging following intraperitoneal injection of D-luciferin (150 mg/kg). Lung metastatic burden was quantified by total photon flux using the Xenogen IVIS imaging system and Living Image software. All animal procedures adhered to institutional ethical guidelines and were approved by the Ethics Committee of Shenzhen Second People’s Hospital.

### Statistics and reproducibility

Data are presented as mean ± standard deviation (SD). Statistical analyses were conducted using GraphPad Prism 8.0 (GraphPad Software) and SPSS 20.0 (IBM). For comparisons between two groups, either a two-tailed Student’s *t*-test or a Mann–Whitney *U*-test was applied, depending on data distribution. For comparisons involving multiple groups, one-way ANOVA followed by Tukey’s multiple-comparisons test was used. A *P*-value of <0.05 was considered statistically significant. All biological experiments were independently performed at least three times unless otherwise stated. Each biological replicate included two technical replicates per measurement. Quantitative data represent the mean ± SD of the biological replicates, as detailed in the corresponding figure legends.

## Results

### Establishment of the ETTAS platform and ortholog screening for translational activation

**Figure 1.**
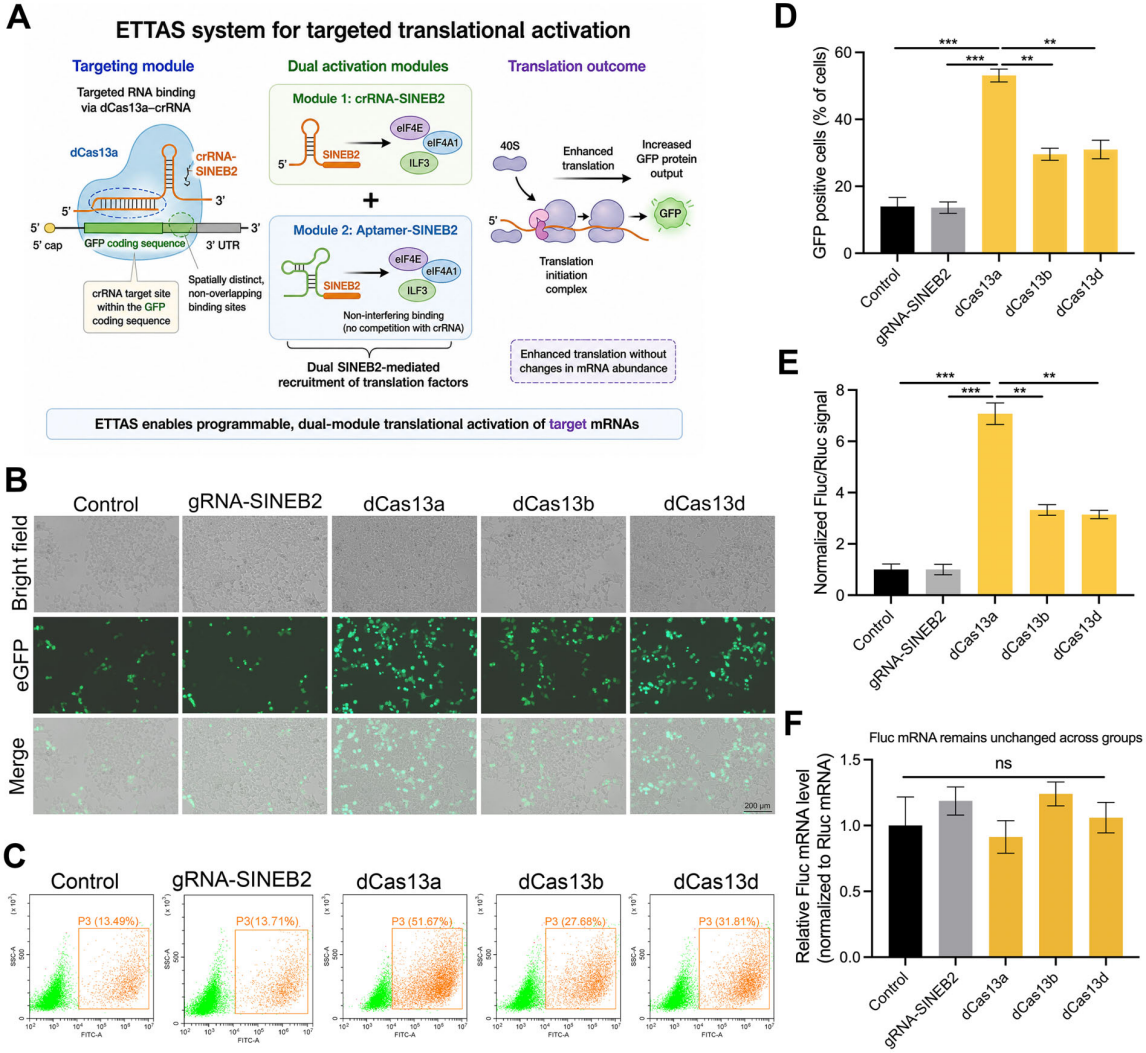
Screening and evaluation of dCas13 orthologs for SINEB2-mediated translational activation. (**A**) Schematic overview of the dCas13-based translational activation platform used to compare orthologs. Each construct contains a crRNA fused with a single SINEB2 element targeting an internal region of the GFP coding sequence (CDS) downstream of the start codon. RNA accessibility and predicted secondary structure were considered during crRNA design to minimize interference with translation initiation and elongation. dCas13 orthologs (Cas13a, Cas13b, and Cas13d) were tested in parallel to identify the most effective scaffold for SINEB2-mediated translational enhancement. (**B**) Representative fluorescence microscopy images of HEK293T cells co-transfected with the GFP reporter and the indicated dCas13-SINEB2 constructs; scale bar: 200 µm. (**C**) Representative flow cytometry plots showing GFP-positive cells in HEK293T cells transfected with the indicated constructs. (**D**) Quantification of GFP-positive cell proportions from flow cytometry analysis. (**E**) Dual-luciferase reporter assay showing translational activation efficiency. Firefly luciferase (Fluc) activity was normalized to Renilla luciferase (Rluc) as an internal control. (**F**) RT-qPCR analysis of relative Fluc mRNA levels normalized to Rluc mRNA across the indicated groups. Data represent mean ± SD from three independent biological replicates, with two technical replicates per biological replicate. Statistical comparisons were performed using one-way ANOVA followed by Tukey’s multiple-comparisons test. Significance levels are indicated as **P < 0.01 and ***P < 0.001.

To establish a programmable RNA-guided translational activation platform, we developed ETTAS, a modular system designed to enhance protein synthesis without altering target mRNA abundance. To move beyond direct crRNA-scaffold optimization, we developed ETTAS as a decoupled modular translational activation system in which target recognition and auxiliary activation are implemented through separate but cooperative RNA modules (Fig. 1A). In this design, dCas13a serves as an RNA-targeting scaffold that positions translation-enhancing SINEB2 elements in proximity to the target transcript while preserving programmable RNA recognition.

To identify an optimal Cas13 scaffold for translational activation, we constructed SINEB2-based systems using dCas13a, dCas13b, and dCas13d orthologs. For all systems, crRNAs were designed to target the same internal region within the GFP coding sequence downstream of the start codon. During crRNA design, local RNA accessibility and predicted secondary structures were explicitly considered using *in silico* folding algorithms. Regions predicted to form highly stable hairpins, extensive double-stranded structures, or extreme GC-rich sequences were avoided to maximize Cas13a binding efficiency. Candidate guides were subsequently validated empirically in dual-luciferase and GFP reporter assays, and the most effective guides were selected for downstream experiments.

HEK293T cells were co-transfected with the indicated dCas13-SINEB2 constructs and a GFP reporter plasmid. Fluorescence microscopy showed that the dCas13b- and dCas13d-based systems increased GFP signal by approximately 2.1–2.5-fold relative to control, whereas the dCas13a-based system achieved a 7.3-fold increase (Fig. 1B). Flow cytometric analysis further confirmed that dCas13a produced the highest proportion of GFP-positive cells among the tested orthologs (Fig. 1C and D). Dual-luciferase assays using a Firefly luciferase (Fluc) reporter with Renilla luciferase (Rluc) normalization similarly demonstrated that dCas13a achieved the strongest reporter activation, with Fluc activity increased 7.3-fold relative to control compared with 2.2–2.5-fold for dCas13b/d (Fig. 1E). RT-qPCR analysis confirmed that Fluc mRNA levels remained largely unchanged across all groups (Fig. 1F), indicating that the observed enhancement resulted primarily from post-transcriptional translational activation rather than changes in transcript abundance. These data further suggest that although the dCas13a–crRNA complex remains bound within the coding sequence, elongating ribosomes can effectively displace or bypass the bound protein, allowing efficient translation without impairing elongation.

Because the number and arrangement of SINEB2 modules may affect RNA folding, stability, or spatial positioning on the target transcript, we evaluated dCas13a constructs containing one, two, or three tandem SINEB2 elements. The single-copy SINEB2 configuration produced the highest translational activation, whereas increasing the number of tandem SINEB2 repeats reduced reporter activity (Supplementary Fig. S2A). Based on these results, the dCas13a scaffold with a single crRNA-fused SINEB2 module was selected for subsequent ETTAS engineering and aptamer-mediated enhancement.

### Identification and characterization of dCas13a-binding aptamers for modular translational activation

Because increasing the copy number of SINEB2 elements within the crRNA scaffold did not enhance translational activation and instead reduced reporter activity (Supplementary Fig. S2A), we adopted a modular strategy to recruit additional translational activation modules independently of the crRNA scaffold. In this design, the dCas13a–crRNA/SINEB2 complex serves as the targeting module, while auxiliary SINEB2 elements can be recruited through aptamer-mediated binding without interfering with crRNA-guided targeting, establishing the conceptual framework for the ETTAS. Accordingly, the goal of aptamer screening was not merely to identify strong dCas13a binders but to identify a binding scaffold compatible with crRNA-guided targeting and suitable for recruitment of an auxiliary SINEB2 module.

**Figure 2.**
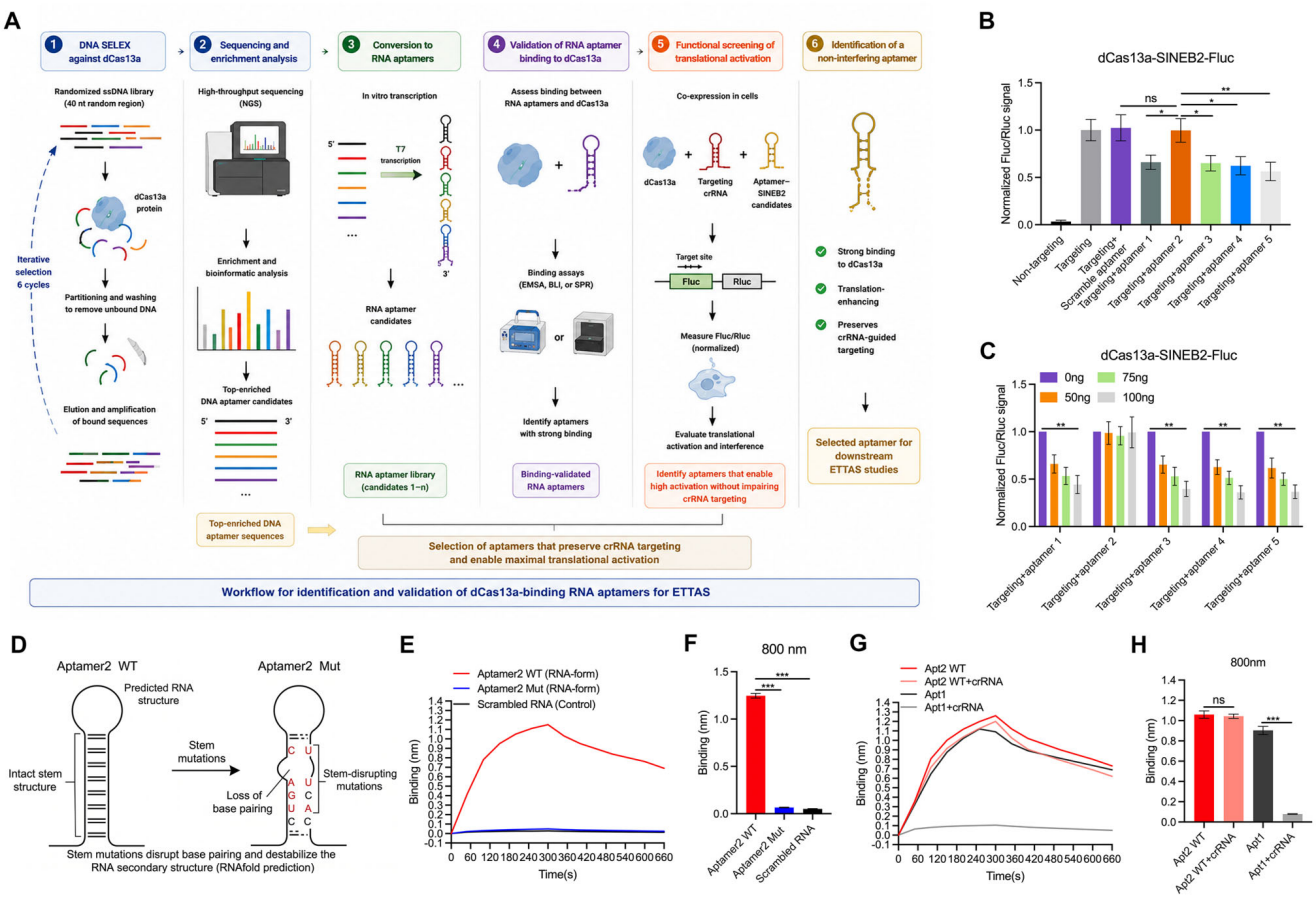
Identification and characterization of dCas13a-binding RNA aptamers for modular translational activation. (**A**) Workflow for selection and validation of dCas13a-binding aptamers. Randomized ssDNA libraries were subjected to iterative SELEX against purified dCas13a protein, followed by sequencing and enrichment analysis. Selected DNA aptamers were converted into RNA-form candidates, evaluated for binding to dCas13a, and functionally screened for compatibility with crRNA-guided translational activation in HEK293T cells using a dual-luciferase reporter system. (**B**) Functional pre-screen of five candidate RNA-form aptamers using the dCas13a–crRNA/SINEB2-Fluc reporter system. HEK293T cells were co-transfected with constructs containing individual aptamer candidates, and dual-luciferase activity was measured. (**C**) Dose-dependent compatibility assay of candidate aptamers. Increasing concentrations of each RNA-form aptamer were co-expressed with dCas13a–crRNA/SINEB2 and Fluc/Rluc reporters to assess compatibility with translational activation and potential functional interference. (**D**) Predicted secondary structures of Aptamer2 WT and the stem-disrupted mutant. (**E**) Representative BLI sensorgrams showing binding of RNA-form Aptamer2 WT, stem-disrupted mutant, and scrambled RNA controls to purified dCas13a. (**F**) Quantification of BLI binding responses at the indicated concentration. (**G**) Comparison of Aptamer2 binding to apo-dCas13a and crRNA-loaded dCas13a. (**H**) Cold-competitor binding assay comparing unlabeled Aptamer2 and scrambled RNA controls. Data represent mean ± SD from three independent biological replicates, with two technical replicates per biological replicate. Statistical comparisons were performed using one-way ANOVA followed by Tukey’s multiple-comparisons test. Significance levels are indicated as *P < 0.05, **P < 0.01, and ***P < 0.001.

To identify suitable nucleic acid aptamers, we performed SELEX using randomized single-stranded DNA libraries for six iterative rounds against purified dCas13a protein. High-throughput sequencing revealed progressive enrichment of specific aptamer sequences and reduced library complexity across rounds, confirming successful convergence of the selection process (Supplementary Fig. S6). All sequences identified from the enriched DNA libraries, including read counts, enrichment ratios, and nucleotide composition, are provided in Supplementary Table S5. Representative enriched candidates were subsequently selected for downstream functional evaluation (Fig. 2A).

For functional compatibility with the RNA-guided translational activation system, the enriched DNA aptamer sequences were transcribed *in vitro* into RNA-form aptamers (Fig. 2A). Predicted secondary structures of the top five candidates revealed distinct stem-loop architectures (Supplementary Fig. S2B). These RNA-form aptamers were individually co-expressed with the dCas13a–crRNA/SINEB2 system in HEK293T cells, and their effects on translational activation were assessed using a dual-luciferase reporter assay. Among the five candidates, Aptamer 2 exhibited minimal interference with reporter activity, whereas Aptamers 1, 3, 4, and 5 partially reduced translational activation (Fig. 2B). Dose–response analysis further confirmed that Aptamer 2 maintained robust reporter activation across increasing concentrations, while the other candidates displayed progressively stronger inhibitory effects (Fig. 2C). These results highlight Aptamer 2 as a non-interfering dCas13a-binding scaffold suitable for modular recruitment of an auxiliary SINEB2 element in the ETTAS platform.

To further characterize aptamer binding properties, we performed concentration-gradient SPR analyses. The measured equilibrium dissociation constants (*K*_D_) for Aptamers 1–5 were 3.684, 2.497, 5.268, 5.910, and 4.722 nM, respectively (Supplementary Fig. S3), indicating that all five candidates exhibited nanomolar-scale binding affinity toward dCas13a. Notably, the extent of functional interference did not strictly correlate with binding affinity, suggesting that functional compatibility depended not only on affinity but also on binding position and steric effects on crRNA-mediated targeting.

To gain structural insight into aptamer–dCas13a interactions, we first examined the predicted secondary structures of the candidate aptamers (Supplementary Fig. S2B). We then generated structural models for docking analysis using Vfold3D and I-TASSER, followed by molecular docking with HDOCK (Supplementary Fig. S2C). Docking analyses suggested that Aptamers 1, 3, 4, and 5 potentially associated with regions proximal to the REC lobe of dCas13a, whereas Aptamer 2 was predicted to bind a spatially distinct region outside the putative crRNA interaction surface. Although these docking models do not define definitive binding interfaces, the predicted binding patterns were broadly consistent with the functional interference profiles observed in reporter assays. The corresponding docking scores and confidence values are summarized in Supplementary Table S4, and the predicted amino acid-nucleotide interaction patterns are shown in Supplementary Fig. S4.

We next experimentally validated the RNA-form binding properties of Aptamer 2 using BLI. RNA-form Aptamer2-WT exhibited robust binding signals toward purified dCas13a, whereas both the stem-mutated Aptamer2 construct and scrambled RNA controls showed minimal binding activity (Fig. 2D–F), indicating that the interaction depended on preservation of the predicted stem-loop structure. Importantly, Aptamer2 retained comparable binding to apo-dCas13a and crRNA-loaded dCas13a complexes, whereas Aptamer1 displayed substantially reduced binding after crRNA loading (Fig. 2G–H), supporting a non-competitive binding mode for Aptamer2. In addition, cold-competitor BLI assays further demonstrated that unlabeled Aptamer2-WT specifically competed for dCas13a binding in a dose-dependent manner, whereas scrambled RNA competitors had minimal effects (Supplementary Fig. S5), confirming sequence-specific recognition.

These computational, biochemical, and functional analyses identified Aptamer2 as a structurally dependent, non-interfering RNA aptamer suitable for modular recruitment of additional SINEB2 translational activation elements in the ETTAS platform.

### Development and validation of the Enhanced Targeted Translational Activation System

**Figure 3.**
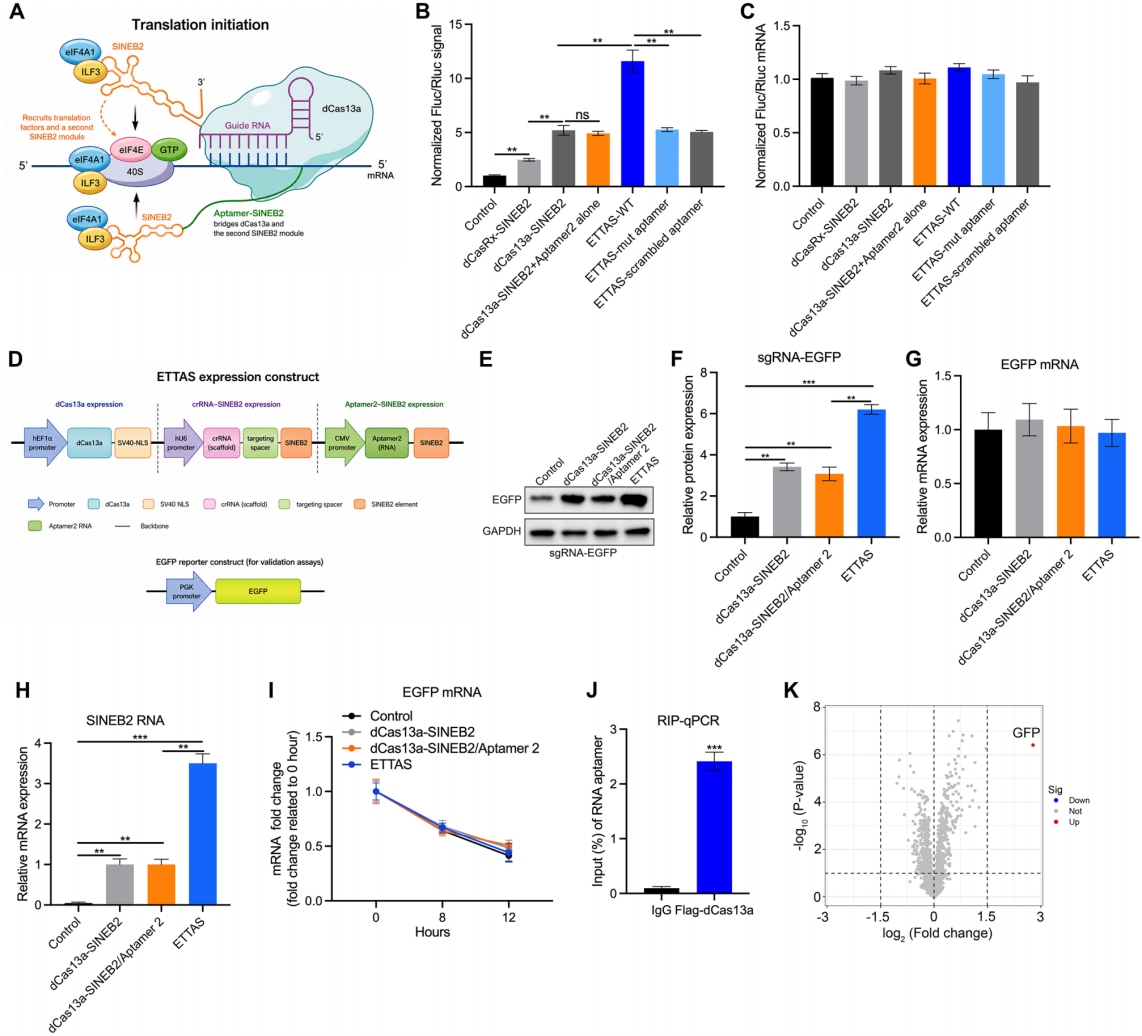
Design and validation workflow of the ETTAS. (**A**) Schematic illustration of the ETTAS mechanism. A crRNA-linked SINEB2 module mediates target recognition, while an Aptamer2–SINEB2 module is recruited to dCas13a through RNA-protein interaction, enabling dual SINEB2-mediated translational enhancement. (**B**) Dual-luciferase reporter assay comparing translational activation by dCasRx–SINEB2, dCas13a–SINEB2, dCas13a–SINEB2 plus Aptamer2 alone, ETTAS-WT, ETTAS-mut aptamer, and ETTAS-scrambled aptamer. Fluc activity was normalized to Rluc. (**C**) RT-qPCR analysis of relative Fluc mRNA levels normalized to Rluc mRNA across the indicated groups. (**D**) Schematic of the ETTAS expression constructs used for EGFP reporter validation. Independent transcription units drive dCas13a, crRNA–SINEB2, and Aptamer2–SINEB2 expression under hEF1α, U6, and CMV promoters, respectively. (**E**) Representative western blot analysis of EGFP protein expression in HEK293T cells transfected with the indicated constructs. GAPDH serves as a loading control. (**F**) Quantification of relative EGFP protein levels from (E). (**G**) RT-qPCR analysis of relative EGFP mRNA levels normalized to GAPDH. (**H**) RT-qPCR quantification of SINEB2 RNA abundance in the indicated groups. (**I**) Actinomycin D chase assay showing EGFP mRNA decay kinetics over time. (**J**) RIP-qPCR assay showing enrichment of Aptamer2 RNA in Flag–dCas13a immunoprecipitates relative to IgG controls. Percent input is plotted. (**K**) Volcano plot from quantitative proteomic analysis comparing ETTAS-targeted cells with non-targeting controls. Proteins meeting the predefined thresholds are highlighted, and GFP is indicated as the most prominently and consistently enriched target protein. Data represent mean ± SD from three independent biological replicates, with two technical replicates per biological replicate, unless otherwise stated. Statistical comparisons were performed using one-way ANOVA followed by Tukey’s multiple-comparisons test, except for panel (J), in which an unpaired two-tailed Student’s *t*-test was used. Asterisks above individual bars indicate comparisons with the corresponding control group, whereas asterisks above connecting lines indicate the indicated pairwise comparisons. Significance levels are indicated as ns, **P < 0.01, and ***P < 0.001.

Having identified Aptamer2 as a binding-validated and functionally non-interfering dCas13a-binding scaffold, we next asked whether it could be used to implement a second, independently recruitable translational activation module. To this end, Aptamer 2 was fused to a second SINEB2 translational activation module, generating a bifunctional RNA scaffold that simultaneously binds dCas13a and recruits an auxiliary SINEB2 element near the target transcript. This design established the ETTAS, in which the dCas13a–crRNA/SINEB2 complex confers target specificity, while the independently expressed Aptamer2–SINEB2 module functions as a secondary enhancer tethered through aptamer–protein interaction (Fig. 3A). Unlike the previously reported dCasRx–SINEB2 architecture [15], in which target recognition and activation are directly coupled within the crRNA scaffold, ETTAS spatially separates these functions, creating a decoupled, modular translational activation framework.

To evaluate translational activation efficiency, HEK293T cells were co-transfected with the corresponding activation constructs and a dual-luciferase reporter. ETTAS significantly enhanced reporter activity, reaching ∼10–12-fold over the control group and outperforming both the original dCas13a–crRNA/SINEB2 and dCas13a–crRNA/SINEB2 + Aptamer2 alone groups (Fig. 3B). Importantly, the “Aptamer2 alone” control, which lacks the fused SINEB2 element, did not significantly increase reporter output relative to dCas13a–crRNA/SINEB2, confirming that Aptamer 2 functions as a recruitment scaffold rather than an independent activator. Mutant or scrambled aptamer controls largely abolished the enhancement, demonstrating that efficient translational activation depends on the aptamer-mediated recruitment mechanism. RT-qPCR analysis showed that Fluc mRNA abundance remained largely unchanged across all groups (Fig. 3C), indicating that the enhanced reporter output is mediated post-transcriptionally.

To further validate the dual-module architecture, independent expression constructs were generated in which dCas13a, crRNA–SINEB2, and Aptamer2–SINEB2 are expressed from separate transcriptional units under hEF1α, U6, and CMV promoters, respectively (Fig. 3D). Using an EGFP reporter, western blot analysis showed that ETTAS substantially increased EGFP protein levels compared with both dCas13a–crRNA/SINEB2 and the Aptamer2-only condition (Fig. 3E and F), producing approximately a twofold enhancement. RT-qPCR confirmed that EGFP mRNA levels remained unchanged (Fig. 3G), demonstrating that ETTAS operates primarily at the translational level.

We next examined whether the additional Aptamer2–SINEB2 module affects SINEB2 RNA abundance. RT-qPCR revealed significantly elevated SINEB2 RNA in ETTAS-expressing cells relative to dCas13a–crRNA/SINEB2 and Aptamer2-only controls (Fig. 3H). Transcriptional inhibition assays using actinomycin D demonstrated that EGFP mRNA decay kinetics were nearly identical across all groups (Fig. 3I), indicating that the dCas13a–crRNA complex, although bound within the coding sequence, can be effectively displaced or bypassed by elongating ribosomes, allowing efficient translation without impeding elongation.

To further characterize the aptamer–dCas13a interaction, RNA immunoprecipitation followed by qPCR (RIP-qPCR) confirmed robust enrichment of Aptamer2 RNA with Flag–dCas13a compared to IgG controls (Fig. 3J). BLI demonstrated direct and specific binding of RNA-form Aptamer2 to dCas13a, while stem-mutant and scrambled RNA controls showed only background binding (Supplementary Fig. S4). Aptamer2 binding remained largely unchanged on apo versus crRNA-loaded dCas13a, indicating a non-competitive recruitment mode (Supplementary Fig. S5). Cold-competitor assays confirmed sequence-specific engagement (Supplementary Fig. S6). Collectively, these data validate that Aptamer2 recruits the auxiliary SINEB2 module without disrupting crRNA-mediated targeting.

Finally, quantitative proteomic analysis comparing ETTAS-targeted cells with non-targeting controls (Fig. 3K) showed that GFP was the only protein consistently and enriched across biological replicates. A predefined fold-change threshold of log_2_ > 1.5 was applied to identify substantial upregulation. While a small number of additional proteins exhibited changes near this threshold, these did not reach statistical significance after correction for multiple testing, and the majority of proteins remained centered near baseline levels. This analysis confirms that ETTAS selectively enhances translation of the intended target with minimal global proteomic perturbation. The combination of a stringent fold-change cutoff and statistical assessment ensures that the observed effects reflect genuine target-specific translational activation rather than experimental noise, supporting both the specificity and functional modularity of the system.

### Validation of ETTAS-mediated activation of endogenous tumor suppressor proteins in bladder cancer cells

To further evaluate the programmability and biological applicability of ETTAS in endogenous settings, we next targeted two clinically relevant tumor suppressor genes, P53 and PTEN, in bladder cancer cells. These targets were selected because they had previously been investigated using first-generation dCasRx–SINEB2 translational activation systems, thereby providing a benchmark for direct comparison with ETTAS [15].

HEK293T-based reporter systems were therefore extended to two bladder cancer cell lines, T24 and 5637, to assess whether ETTAS could efficiently enhance endogenous protein expression across distinct cellular contexts. Cells were transfected with ETTAS constructs, dCasRx–SINEB2 comparator systems, or corresponding non-targeting controls.

**Figure 4.**
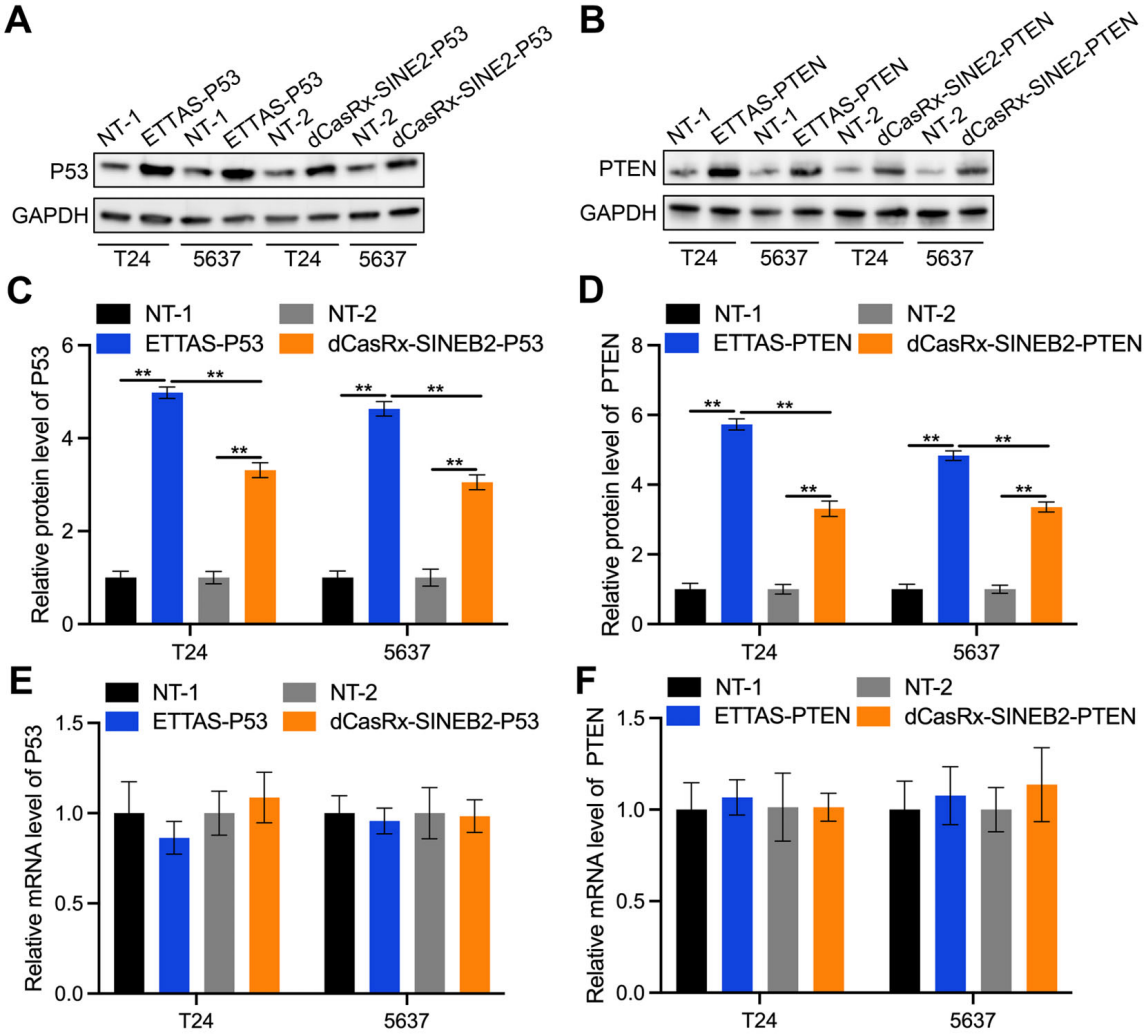
Evaluation of the ETTAS in activating endogenous protein expression in bladder cancer cell lines. (**A** and **B**) Western blot analysis of P53 and PTEN protein levels in T24 and 5637 bladder cancer cells transfected with ETTAS, dCasRx–SINEB2, and their respective negative controls. Two independent non-targeting guide RNAs (NT-1 and NT-2) were included as negative controls to account for potential sequence-dependent effects of crRNA expression. GAPDH was used as a loading control. (**C** and **D**) Quantification of relative protein expression levels of P53 and PTEN, showing the effect of ETTAS compared with dCasRx–SINEB2 and matched NT-gRNA controls in both cell lines. (**E** and **F**) RT-qPCR analysis of relative mRNA levels of P53 and PTEN, demonstrating that ETTAS did not significantly alter mRNA abundance compared with matched NT-gRNA controls, indicating that the observed effects occur predominantly at the post-transcriptional level. For all experiments shown in this figure, a NT-gRNA was included as the negative control, and comparisons were performed between targeting crRNA and matched NT-gRNA conditions under otherwise identical experimental settings, ensuring that the observed effects are specific to the guide RNA sequence. Data represent mean ± SD from three independent biological replicates, with two technical replicates per biological replicate. Statistical comparisons were performed using one-way ANOVA followed by Tukey’s multiple-comparisons test. Asterisks above connecting lines indicate the indicated pairwise comparisons. Significance levels are indicated as **P < 0.01.

Western blot analysis showed that ETTAS induced stronger upregulation of endogenous P53 and PTEN protein expression than the dCasRx–SINEB2 system in both T24 and 5637 cells (Fig. 4A–D). Quantitative analysis confirmed that ETTAS consistently achieved the highest protein induction efficiency across both endogenous targets and both bladder cancer cell lines, supporting the robustness and programmability of the platform.

To determine whether the observed increase in protein abundance was accompanied by changes at the transcript level, we next quantified endogenous P53 and PTEN mRNA expression using RT-qPCR. No significant differences in P53 or PTEN mRNA abundance were observed among the tested groups (Fig. 4E and F), despite the marked increase in protein expression. These findings are consistent with the results obtained in reporter systems and further support a predominantly translational rather than transcriptional mechanism underlying ETTAS-mediated gene activation.

Together, these results demonstrate that ETTAS can efficiently and programmably enhance endogenous tumor suppressor protein expression across multiple cellular contexts while minimally affecting target mRNA abundance, highlighting its potential utility as a modular RNA-guided translational activation platform.

### Functional validation of ETTAS-mediated tumor suppressor activation in bladder cancer cells

To determine whether ETTAS-mediated translational activation of endogenous tumor suppressor genes produces functional antitumor effects, we evaluated cellular proliferation and apoptosis in bladder cancer cells following activation of P53 or PTEN. T24 and 5637 bladder cancer cells were transfected with ETTAS–P53, ETTAS–PTEN, dCasRx–SINEB2 controls, or matched non-targeting controls, followed by functional analyses.

**Figure 5.**
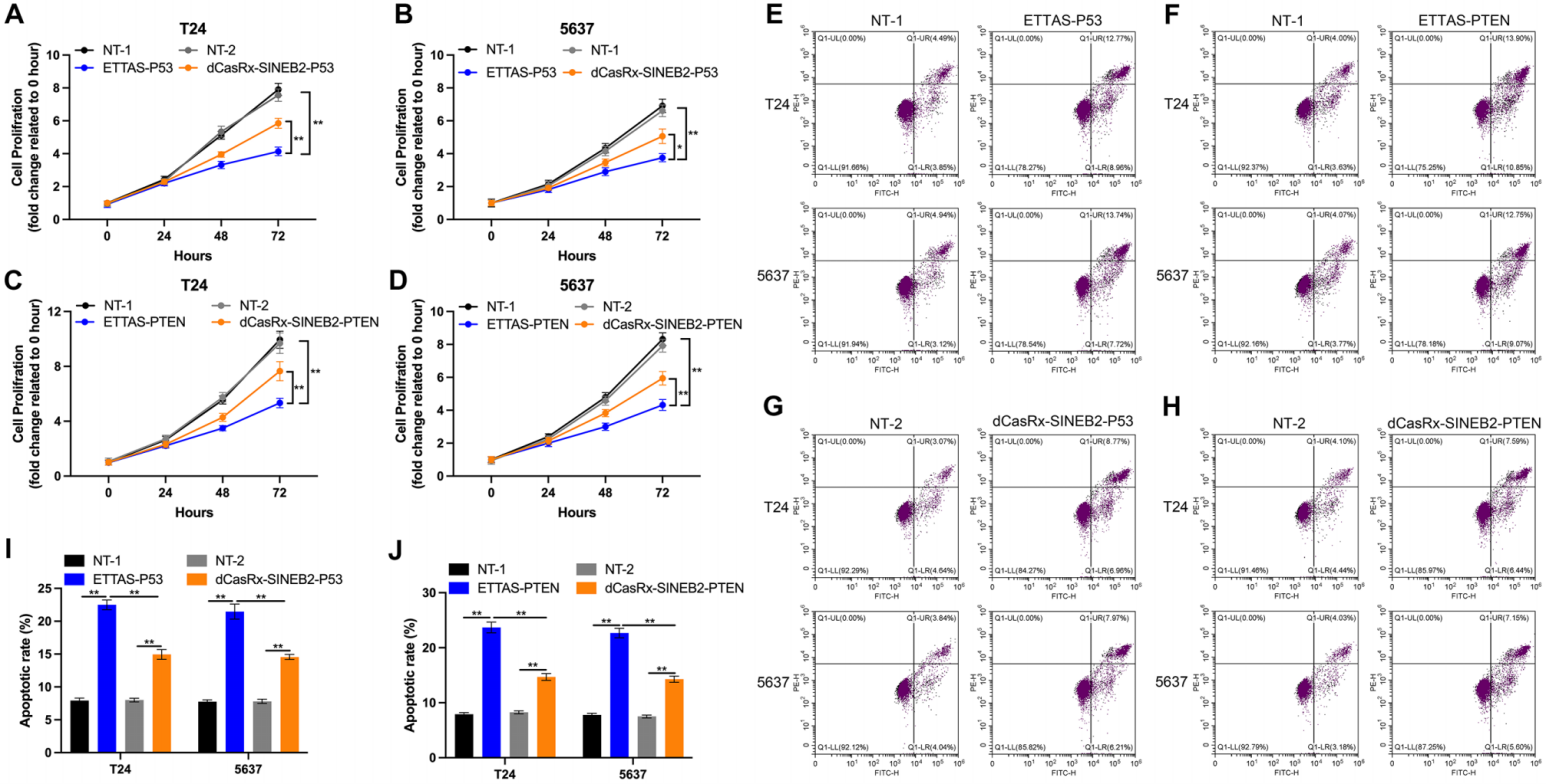
Evaluation of the effects of ETTAS-mediated activation of P53 and PTEN on cell proliferation and apoptosis in bladder cancer cell lines. (**A–D**) CCK-8 assay results showing the proliferation rates of T24 and 5637 bladder cancer cells following transfection with ETTAS constructs or dCasRx–SINEB2 targeting P53 (A and B) and PTEN (C and D). Two independent non-targeting guide RNAs (NT-1 and NT-2) were included as negative controls. Cell proliferation was compared between targeting crRNA and matched NT-gRNA conditions over a period of 72 h. (**E–H**) Representative Annexin V-FITC/PI flow cytometry plots of T24 and 5637 cells transfected with ETTAS or dCasRx–SINEB2 constructs targeting P53 or PTEN, together with their matched NT-gRNA controls (NT-1 or NT-2). Cells were classified as viable (Annexin V−/PI−), early apoptotic (Annexin V+/PI−), late apoptotic/secondary necrotic (Annexin V+/PI+), or necrotic (Annexin V−/PI+). (**I** and **J**) Quantification of apoptotic rates derived from the flow cytometry analyses shown in panels (E–H). The total apoptotic fraction was calculated as the sum of early apoptotic and late apoptotic cells (Annexin V+/PI − plus Annexin V+/PI+). Data are presented as mean ± SD from three independent biological replicates. For all experiments shown in this figure, NT-gRNAs were included as negative controls, and comparisons were performed between targeting crRNA and matched NT-gRNA conditions under otherwise identical experimental settings, ensuring that the observed effects are specific to the guide RNA sequence. Data represent mean ± SD from three independent biological replicates, with two technical replicates per biological replicate. Statistical comparisons were performed using one-way ANOVA followed by Tukey’ s multiple-comparisons test. Asterisks above connecting lines indicate the indicated pairwise comparisons. Significance levels are indicated as *P < 0.05, **P < 0.01.

Cell proliferation assays showed that activation of either P53 or PTEN by ETTAS suppressed proliferation of both T24 and 5637 cells compared with the corresponding non-targeting controls and dCasRx–SINEB2 groups (Fig. 5A–D). Notably, the inhibitory effect of ETTAS was consistently stronger than that observed with the original dCasRx–SINEB2 system, indicating that the enhanced translational activation capacity of ETTAS translated into improved functional tumor suppressive activity.

Apoptosis was assessed using Annexin V/PI flow cytometry. ETTAS-mediated activation of P53 or PTEN increased apoptotic cell populations in both bladder cancer cell lines (Fig. 5E–H). Quantitative analysis further confirmed elevated apoptotic rates in the ETTAS groups relative to both non-targeting and dCasRx–SINEB2 controls (Fig. 5I and J).

To further characterize the downstream molecular consequences of ETTAS-mediated P53 activation, we examined expression of canonical P53 target proteins, including P21, PUMA, and BAX. Western blot analysis showed that ETTAS–P53 induced substantially stronger upregulation of P53 and its downstream effectors compared with both the original dCas13a–SINEB2 and dCasRx–SINEB2 systems (Supplementary Fig. S7A and B). Importantly, mutation of the Aptamer2 module partially attenuated this enhancement effect, supporting the functional contribution of the Aptamer2–SINEB2 recruitment mechanism. RT-qPCR analysis confirmed that P53 mRNA abundance remained largely unchanged across all groups (Supplementary Fig. S7C), consistent with post-transcriptional regulation.

These data collectively indicate that the observed suppression of proliferation and induction of apoptosis are consistent with enhanced expression of P53 and PTEN and associated downstream pathway activation, supporting target-specific post-transcriptional activation by ETTAS. Together, these findings demonstrate that ETTAS-mediated translational activation not only enhances endogenous tumor suppressor protein expression but also produces robust downstream biological effects, including inhibition of bladder cancer cell proliferation and induction of apoptosis.

### AAV-mediated *in vivo* delivery of ETTAS suppresses bladder tumor growth through translational activation of P53

**Figure 6.**
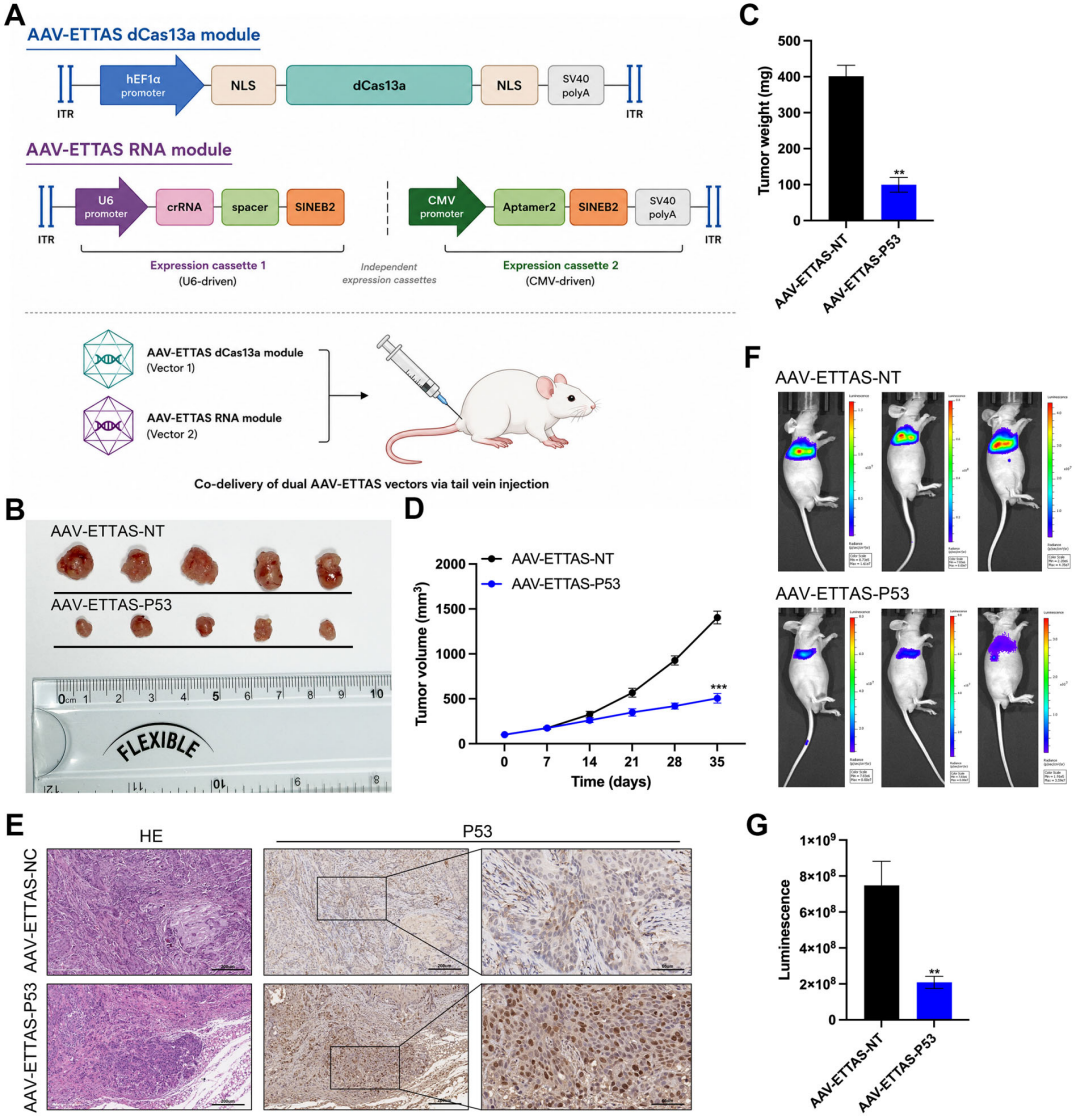
*In vivo* evaluation of the ETTAS system in tumor growth suppression and metastasis inhibition. (**A**) Schematic representation of the dual-vector AAV-ETTAS delivery system. The AAV-ETTAS dCas13a module encodes dCas13a under the hEF1α promoter, whereas the AAV-ETTAS RNA module encodes crRNA–SINEB2 and Aptamer2–SINEB2 under U6 and CMV promoters, respectively. The two vectors were co-administered via tail vein injection. (**B**) Representative images of subcutaneous xenograft tumors collected from mice treated with AAV-ETTAS-P53 or the non-targeting control (AAV-ETTAS-NT). (**C**) Quantification of tumor weights at the experimental endpoint. (**D**) Tumor growth curves showing tumor volume progression over time in the indicated treatment groups. (**E**) Representative H&E staining and IHC analysis of P53 expression in xenograft tumor sections from the indicated groups. (**F**) Representative bioluminescence images from the lung metastasis model established using luciferase-labeled bladder cancer cells following systemic administration of dual-AAV vectors expressing either ETTAS-P53 or the non-targeting control. (**G**) Quantification of lung bioluminescence signals in the indicated groups. For all *in vivo* experiments, the non-targeting control (AAV-ETTAS-NT) preserved the same dual-vector delivery framework and differed from the targeting condition only in the guide RNA sequence. Data represent mean ± SD from five biological replicates per group. Statistical comparisons were performed using unpaired two-tailed Student’s *t*-test. Significance levels are indicated as **P < 0.01 and ***P < 0.001.

To evaluate the therapeutic potential of ETTAS *in vivo*, we developed a dual-AAV delivery strategy for systemic administration of the translational activation system (Fig. 6A). The AAV-ETTAS platform consisted of two separate vectors: an AAV-dCas13a module expressing dCas13a under the control of the hEF1α promoter, and an AAV-RNA module containing both the U6-driven crRNA–SINEB2 cassette and the CMV-driven Aptamer2–SINEB2 cassette. A non-targeting control (AAV-ETTAS-NT), in which the guide RNA sequence does not target the intended transcript, was included as a negative control. The two vectors were co-delivered via tail vein injection to establish systemic expression of ETTAS components in tumor-bearing mice.

To determine whether ETTAS-mediated translational activation could inhibit tumor progression *in vivo*, xenograft-bearing mice were treated with either AAV-ETTAS-P53 or AAV-ETTAS-NT. At the experimental endpoint, tumors harvested from the AAV-ETTAS-P53 group were visibly smaller than those from control animals (Fig. 6B), and tumor weights were reduced (Fig. 6C). Longitudinal monitoring demonstrated that AAV-ETTAS-P53 suppressed tumor growth over the treatment course, resulting in lower tumor volumes compared with the control group (Fig. 6D).

Histopathological examination by H&E staining revealed increased necrotic and degenerative regions in tumors treated with AAV-ETTAS-P53 (Fig. 6E). Immunohistochemistry confirmed enhanced P53 protein expression in the treatment group relative to controls, consistent with successful *in vivo* translational activation of endogenous P53 (Fig. 6E).

Bioluminescence imaging was performed to noninvasively monitor metastatic progression. Representative images showed reduced lung-associated luminescence in mice receiving AAV-ETTAS-P53 compared with control animals, and quantitative analysis confirmed a significant decrease in luminescence intensity (Fig. 6F and G). Together with the increased P53 protein expression detected by IHC, these findings support functional activity of the delivered ETTAS system *in vivo*.

These results demonstrate that systemic AAV-mediated delivery of ETTAS enables functional *in vivo* translational activation of endogenous P53 and exerts antitumor and anti-metastatic effects in bladder cancer models. We note that the absolute *in vivo* stoichiometry of the crRNA–SINEB2 and Aptamer2–SINEB2 RNA components was not directly quantified in the current study and will require further investigation in future optimization of the AAV-based ETTAS platform.

## Discussion

In this study, we systematically optimized Cas13-mediated translational activation and developed a modular RNA-guided platform, termed ETTAS, for enhanced post-transcriptional protein upregulation. By combining a crRNA-linked SINEB2 activation module with an independently recruited aptamer–SINEB2 enhancer, ETTAS achieved substantially stronger translational activation than the previously reported dCasRx–SINEB2 system [15] while maintaining transcript specificity and minimal effects on target mRNA abundance. Importantly, ETTAS effectively activated endogenous tumor suppressor genes both *in vitro* and *in vivo*, highlighting its potential as a programmable therapeutic platform for precision translational regulation.

A major finding of this study is that different Cas13 orthologs exhibit markedly distinct capacities for translational activation. Among the systems evaluated, dCas13a consistently outperformed dCas13b, dCas13d, and dCasRx-based architectures in reporter activation assays. Although the precise structural basis underlying these differences was not directly dissected, our results suggest that the architectural organization of Cas13 proteins may critically influence compatibility with translational activation modules. Compared with other orthologs, Cas13a may provide a more permissive scaffold for recruitment or spatial positioning of SINEB2-associated translation factors. These observations are consistent with prior studies showing functional diversity among Cas13 orthologs and the versatility of catalytically inactive CRISPR–Cas effectors as programmable regulatory scaffolds [3, 4, 6, 21, 22], and suggest that Cas13 orthologs should be evaluated individually in post-transcriptional engineering applications.

An additional important observation was that simple tandem expansion of SINEB2 elements failed to improve translational activation and instead reduced reporter output. This finding suggests that translational enhancement mediated by SINEB2 is not linearly scalable through direct repetition of activation elements. One possible explanation is that excessive RNA length or repetitive secondary structures may impair RNA folding, reduce transcript stability, or interfere with assembly of functional ribonucleoprotein complexes. Alternatively, closely linked repetitive SINEB2 modules may create steric or topological constraints that negatively affect translational initiation factor recruitment. Given that SINEB2-mediated translational activation depends on RNA architecture and interaction with translation-associated factors [10, 12–14], these results indicate that increasing translational potency requires more sophisticated architectural optimization than merely increasing enhancer copy number.

To address this issue, we introduced an aptamer-mediated recruitment strategy that spatially separates activation modules while maintaining proximity to the target transcript. Using SELEX screening, we identified multiple Cas13a-binding aptamers and subsequently selected Aptamer2 because it remained compatible with crRNA-guided targeting while retaining direct binding activity. Importantly, the functional behavior of Aptamer2 differed substantially from that of other aptamers despite their broadly comparable binding affinities, indicating that binding geometry and structural compatibility may be more important determinants of functionality than affinity alone. This observation highlights an important design principle for programmable RNA scaffolds: auxiliary RNA-binding modules must preserve the conformational and functional integrity of the underlying CRISPR ribonucleoprotein complex, consistent with prior CRISPR–aptamer engineering studies [16, 20].

The conversion of the SELEX-derived DNA aptamer into an RNA-form aptamer represented another important aspect of the study. Because RNA aptamers are more compatible with intracellular transcription systems, this conversion was essential for implementing a fully genetically encoded activation platform. Importantly, the RNA-form Aptamer2 was experimentally validated to bind dCas13a directly and specifically while remaining compatible with crRNA loading, as supported by BLI assays, RIP-qPCR, and competition analyses. Together with molecular docking predictions, these findings are consistent with a non-competitive binding mode in which Aptamer2 associates with dCas13a without disrupting the crRNA recognition interface. This property enabled modular recruitment of a second SINEB2 activation element without compromising target recognition.

Notably, ETTAS differs conceptually from our previously reported dCasRx–SINEB2 architecture [15]. In the original dCasRx–SINEB2 system, the translational activation element was directly embedded within the targeting crRNA scaffold, thereby coupling target recognition and translational enhancement within a single RNA component. In contrast, ETTAS establishes a decoupled modular architecture in which an independently expressed aptamer–SINEB2 module is recruited through non-competitive aptamer–dCas13a interaction. This separation enables flexible and orthogonal recruitment of auxiliary translational activation modules without directly altering the crRNA scaffold itself. The present study further demonstrates that simple tandem expansion of SINEB2 elements is insufficient to improve activation efficiency, whereas spatially separated aptamer-mediated recruitment successfully enhances translational output. Thus, ETTAS should not be viewed merely as an optimized version of previous systems, but rather as a modular translational engineering framework for programmable post-transcriptional regulation. In this sense, the earlier dCasRx–SINEB2 system may remain advantageous when compactness and delivery simplicity are prioritized, whereas ETTAS provides a more flexible architecture when stronger activation and modular expandability are required.

Based on these findings, we established ETTAS as a dual-module translational activation platform. Compared with the previously reported dCasRx–SINEB2 system, ETTAS produced substantially stronger translational activation while preserving the characteristic post-transcriptional mechanism of action. Multiple lines of evidence support this conclusion. First, target mRNA abundance remained largely unchanged across groups. Second, transcriptional inhibition experiments showed no detectable alteration in mRNA degradation kinetics. Third, proteomic analysis revealed highly selective upregulation of the intended target protein with minimal global proteome perturbation. Together, these findings indicate that ETTAS primarily enhances translation rather than transcription or RNA stability.

Notably, ETTAS showed robust activity on endogenous tumor suppressor genes. Activation of P53 and PTEN in bladder cancer cells resulted in marked inhibition of proliferation and increased apoptosis, indicating that the system can effectively modulate biologically meaningful signaling pathways. Compared with the previously developed dCasRx–SINEB2 platform, ETTAS consistently achieved stronger protein induction and greater phenotypic effects. Because many clinically relevant genes are constrained by insufficient protein expression rather than irreversible genetic defects, programmable translational activation systems such as ETTAS may offer therapeutic advantages over permanent genome editing approaches [1, 20]. In particular, transient and reversible enhancement of endogenous protein synthesis could reduce risks associated with DNA modification while preserving native transcriptional regulation.

The *in vivo* AAV delivery experiments further support the translational potential of this platform. Despite the packaging limitations associated with AAV systems, we successfully implemented a dual-vector AAV-ETTAS architecture capable of systemic delivery and endogenous P53 activation in tumor-bearing mice. AAV-mediated ETTAS delivery significantly suppressed tumor growth and reduced tumor burden in xenograft models, demonstrating that programmable translational activation can be achieved *in vivo* using clinically relevant delivery modalities. These findings expand the potential therapeutic scope of RNA-guided translational engineering.

However, the increased modularity of ETTAS introduces additional architectural complexity compared with earlier single-component dCasRx–SINEB2 systems. In particular, the current implementation requires independent expression of the aptamer–SINEB2 module and, for *in vivo* delivery, a dual-AAV configuration. Although this modular design provides greater flexibility and enhanced activation capacity, future optimization toward more compact architectures, minimized RNA scaffolds, or single-vector delivery systems will likely further improve translational applicability. We therefore view the increased complexity not as a trivial extension, but as the engineering tradeoff required to achieve independently recruitable auxiliary activation.

Several limitations of this study should also be acknowledged. First, although docking and biochemical assays support the proposed aptamer-binding model, the precise structural interface between Aptamer 2 and dCas13a remains unresolved. High-resolution structural studies such as cryo-electron microscopy may further clarify the molecular basis of non-competitive aptamer binding. Second, although proteomic analyses suggested limited off-target effects under the conditions tested, broader transcriptome-wide and long-term safety evaluations will be necessary before therapeutic translation. Third, the current study primarily focused on translational activation in cancer models; whether ETTAS can be generalized to other disease contexts or primary cell systems remains to be determined. Finally, optimization of delivery strategies, vector size, and tissue specificity will likely further improve *in vivo* applicability.

Furthermore, the modular architecture of ETTAS may facilitate future integration of additional programmable RNA regulatory modules beyond SINEB2, including ligand-responsive aptamers, conditional translational regulators, or combinatorial post-transcriptional effectors. This flexibility distinguishes ETTAS from fixed single-scaffold translational activation systems and expands the design space for programmable RNA engineering.

In conclusion, this study establishes ETTAS as a modular and programmable platform for RNA-guided translational activation. By integrating aptamer-mediated recruitment with SINEB2-based translational enhancement, ETTAS overcomes key limitations of previous Cas13-mediated activation systems and enables robust upregulation of endogenous proteins without altering target mRNA abundance. Together, these findings establish ETTAS as a modular RNA-guided translational engineering framework that enables programmable and scalable post-transcriptional control of endogenous protein synthesis.

## Supplementary Material

gkag692_Supplemental_Files

## Data Availability

The original western blot raw gel images generated in this study have been deposited in Zenodo and are publicly available at https://doi.org/10.5281/zenodo.20849391. All data supporting the findings of this study are included within the article and its Supplementary Data files.
