# Supplementary material for: ETTAS: a modular aptamer-recruited platform for programmable translational activation
Source: Nucleic Acids Res. 2026 Jul 25;54(14):gkag692. doi: 10.1093/nar/gkag692 (PMC13401040; doi:10.1093/nar/gkag692)
Supplement: gkag692_Supplemental_Files [file gkag692_supplemental_files.zip › Supplementary Materials.docx]

**Supplementary Table.1**

**Maps of plasmids used in this study.**

| pHS-AVC-1001 | pZDonor-PGK-EGFP |
| --- | --- |
| pHS-AVC-1002 | hU6-sgRNA-EGFP-SINEB2-hEF1a-dCas13a-NES |
| pHS-AVC-1003 | hU6-sgRNA-EGFP-SINEB2-hEF1a-dCas13b-NES |
| pHS-AVC-1004 | hU6-sgRNA-EGFP-SINEB2-hEF1a-dCas13d-NES |
| pHS-AVC-1005 | hU6-sgRNA-NC-SINEB2-NES |
| pHS-AVC-1006 | hU6-sgRNA-Fluc-SINEB2-hEF1a-dCas13a-NES |
| pHS-AVC-1007 | hU6-sgRNA-Fluc-SINEB2-hEF1a-dCas13b-NES |
| pHS-AVC-1008 | hU6-sgRNA-Fluc-SINEB2-hEF1a-dCas13d-NES |
| pHS-AVC-1009 | hU6-sgRNA-EGFP-SINEB2-hEF1a-dCas13a-NES-CMV-aptamer2-SINEB2 |
| pHS-AVC-1010 | hU6-sgRNA-P53-SINEB2-hEF1a-dCas13a-NES-CMV-aptamer2-SINEB2 |
| pHS-AVC-1011 | hU6-sgRNA-PTEN-SINEB2-hEF1a-dCas13a-NES-CMV-aptamer2-SINEB2 |

**Supplementary Table.2**

Plasmids sequence

pHS-AVC-1001 pZDonor-PGK-EGFP

AATTCCACGGGGTTGGGGTTGCGCCTTTTCCAAGGCAGCCCTGGGTTTGCGCAGGGACGCGGCTGCTCTGGGCGTGGTTCCGGGAAACGCAGCGGCGCCGACCCTGGGTCTCGCACATTCTTCACGTCCGTTCGCAGCGTCACCCGGATCTTCGCCGCTACCCTTGTGGGCCCCCCGGCGACGCTTCCTGCTCCGCCCCTAAGTCGGGAAGGTTCCTTGCGGTTCGCGGCGTGCCGGACGTGACAAACGGAAGCCGCACGACTCACTAGTACCCTCGCAGACGGACAGCGCCAGGGAGCAATGGCAGCGCGCCGACCGCGATGGGCTGTGGCCAATAGCGGCTGCTCAGCAGGGCGCGCCGAGAGCAGCGGCCGGGAAGGGACGGTGCGGGAGGCGGGGTGTGGGGCGGTAGTGTGGGCCCTGTTCCTGCCCGCGCGGTGTTCCGCATTCTGCAAGCCTCCGGAGCGCACGTCGGCAGTCGGCTCCCTCGTTGACCGAATCACCGACCTCTCTCCCCAGGGGGATCCACCGGTTTGTCGACAAGCTTGGTGGCGGCTTAACTAGTTAAGGGCCCGGCGCGCCTAAGGTACCCCCGGGTAACTGATCATAATTCGACCCAAGTTTGTACAAAAAAGCAGGCTGATTACCGGTCGCCACCATGGTGAGCAAGGGCGAGGAGCTGTTCACCGGGGTGGTGCCCATCCTGGTCGAGCTGGACGGCGACGTAAACGGCCACAAGTTCAGCGTGTCCGGCGAGGGCGAGGGCGATGCCACCTACGGCAAGCTGACCCTGAAGTTCATCTGCACCACCGGCAAGCTGCCCGTGCCCTGGCCCACCCTCGTGACCACCCTGACCTACGGCGTGCAGTGCTTCAGCCGCTACCCCGACCACATGAAGCAGCACGACTTCTTCAAGTCCGCCATGCCCGAAGGCTACGTCCAGGAGCGCACCATCTTCTTCAAGGACGACGGCAACTACAAGACCCGCGCCGAGGTGAAGTTCGAGGGCGACACCCTGGTGAACCGCATCGAGCTGAAGGGCATCGACTTCAAGGAGGACGGCAACATCCTGGGGCACAAGCTGGAGTACAACTACAACAGCCACAACGTCTATATCATGGCCGACAAGCAGAAGAACGGCATCAAGGTGAACTTCAAGATCCGCCACAACATCGAGGACGGCAGCGTGCAGCTCGCCGACCACTACCAGCAGAACACCCCCATCGGCGACGGCCCCGTGCTGCTGCCCGACAACCACTACCTGAGCACCCAGTCCGCCCTGAGCAAAGACCCCAACGAGAAGCGCGATCACATGGTCCTGCTGGAGTTCGTGACCGCCGCCGGGATCACTCTCGGCATGGACGAGCTGTACAAGTAA

pHS-AVC-1002 hU6-sgRNA-EGFP-SINEB2-hEF1a-dCas13a-NES:

*CAGGGTAATTCGGTCAAGCCTTGCCTTGTTGTAGCTTAAATTTTGCTCGCGCACTACTCAGCGACCTCCAACACACAAGCAGGGAGCAGATACTGGCTTAACTATGCGGCATCAGAGCAGATTGTACTGAGAGTGCACCATAGGGGATCGGGAGATCTCCCGATCCGTCGACGTCAGGTGGCACTTTTCGGGGAAATGTGCGCGGAACCCCTATTTGTTTATTTTTCTAAATACATTCAAATATGTATCCGCTCATGAGACAATAACCCTGATAAATGCTTCAATAATATTGAAAAAGGAAGAGTATGAGTATTCAACATTTCCGTGTCGCCCTTATTCCCTTTTTTGCGGCATTTTGCCTTCCTGTTTTTGCTCACCCAGAAACGCTGGTGAAAGTAAAAGATGCTGAAGATCAGTTGGGTGCACGAGTGGGTTACATCGAACTGGATCTCAACAGCGGTAAGATCCTTGAGAGTTTTCGCCCCGAAGAACGTTTTCCAATGATGAGCACTTTTAAAGTTCTGCTATGTGGCGCGGTATTATCCCGTATTGACGCCGGGCAAGAGCAACTCGGTCGCCGCATACACTATTCTCAGAATGACTTGGTTGAGTACTCACCAGTCACAGAAAAGCATCTTACGGATGGCATGACAGTAAGAGAATTATGCAGTGCTGCCATAACCATGAGTGATAACACTGCGGCCAACTTACTTCTGACAACGATCGGAGGACCGAAGGAGCTAACCGCTTTTTTGCACAACATGGGGGATCATGTAACTCGCCTTGATCGTTGGGAACCGGAGCTGAATGAAGCCATACCAAACGACGAGCGTGACACCACGATGCCTGTAGCAATGGCAACAACGTTGCGCAAACTATTAACTGGCGAACTACTTACTCTAGCTTCCCGGCAACAATTAATAGACTGGATGGAGGCGGATAAAGTTGCAGGACCACTTCTGCGCTCGGCCCTTCCGGCTGGCTGGTTTATTGCTGATAAATCTGGAGCCGGTGAGCGTGGGTCACGCGGTATCATTGCAGCACTGGGGCCAGATGGTAAGCCCTCCCGTATCGTAGTTATCTACACGACGGGGAGTCAGGCAACTATGGATGAACGAAATAGACAGATCGCTGAGATAGGTGCCTCACTGATTAAGCATTGGTAACTGTCAGACCAAGTTTACTCATATATACTTTAGATTGATTTAAAACTTCATTTTTAATTTAAAAGGATCTAGGTGAAGATCCTTTTTGATAATCTCATGACCAAAATCCCTTAACGTGAGTTTTCGTTCCACTGAGCGTCAGACCCCGTAGAAAAGATCAAAGGATCTTCTTGAGATCCTTTTTTTCTGCGCGTAATCTGCTGCTTGCAAACAAAAAAACCACCGCTACCAGCGGTGGTTTGTTTGCCGGATCAAGAGCTACCAACTCTTTTTCCGAAGGTAACTGGCTTCAGCAGAGCGCAGATACCAAATACTGTTCTTCTAGTGTAGCCGTAGTTAGGCCACCACTTCAAGAACTCTGTAGCACCGCCTACATACCTCGCTCTGCTAATCCTGTTACCAGTGGCTGCTGCCAGTGGCGATAAGTCGTGTCTTACCGGGTTGGACTCAAGACGATAGTTACCGGATAAGGCGCAGCGGTCGGGCTGAACGGGGGGTTCGTGCACACAGCCCAGCTTGGAGCGAACGACCTACACCGAACTGAGATACCTACAGCGTGAGCTATGAGAAAGCGCCACGCTTCCCGAAGGGAGAAAGGCGGACAGGTATCCGGTAAGCGGCAGGGTCGGAACAGGAGAGCGCACGAGGGAGCTTCCAGGGGGAAACGCCTGGTATCTTTATAGTCCTGTCGGGTTTCGCCACCTCTGACTTGAGCGTCGATTTTTGTGATGCTCGTCAGGGGGGCGGAGCCTATGGAAAAACGCCAGCAACGCGGCCTTTTTACGGTTCCTGGCCTTTTGCTGGCCTTTTGCTCACATGTTCTTTATTACCCTGTTATCCCTAACCGGTTCACCAATTGTATCCGATATCAACTTTGTATAGAAAAGTTGGCTCCGAATTTCTCGAGGAATTCGGCAGGAAGAGGGCCTATTTCCCATGATTCCTTCATATTTGCATATACGATACAAGGCTGTTAGAGAGATAATTAGAATTAATTTGACTGTAAACACAAAGATATTAGTACAAAATACGTGACGTAGAAAGTAATAATTTCTTGGGTAGTTTGCAGTTTTAAAATTATGTTTTAAAATGGACTATCATATGCTTACCGTAACTTGAAAGTATTTCGATTTCTTGGCTTTATATATCTTGTGGAAAGGACGAAACACCGCACTAGTGCGAATTTGCACTAGTCTAAAACCGCCCTTGCTCACCATGGTGGCCAGTGCTAGAGGAGGTCAGAAGAGGGCATTGGATCCCCCAGAACTGGAGTTATACGGTAACCTCGTGGTGGTTGTGAACCACCATGTGGATGGATATTGAGTTCCAAACACTGGTCCTGTGCAAGAGCATCCAGTGCTCTTAAGTGCTGAGCCATCTCTTTAGCTCCTTTTTTTAAGCTTGGCTCCGGTGCCCGTCAGTGGGCAGAGCGCACATCGCCCACAGTCCCCGAGAAGTTGTGGGGAGGGGTCGGCAATTGAACCGGTGCCTAGAGAAGGTGGCGCGGGGTAAACTGGGAAAGTGATGTCGTGTACTGGCTCCGCCTTTTTCCCGAGGGTGGGGGAGAACCGTATATAAGTGCAGTAGTCGCCGTGAACGTTCTTTTTCGCAACGGGTTTGCCGCCAGAACACAGGTAAGTGCCGTGTGTGGTTCCCGCGGGCCTGGCCTCTTTACGGGTTATGGCCCTTGCGTGCCTTGAATTACTTCCACCTGGCTGCAGTACGTGATTCTTGATCCCGAGCTTCGGGTTGGAAGTGGGTGGGAGAGTTCGAGGCCTTGCGCTTAAGGAGCCCCTTCGCCTCGTGCTTGAGTTGAGGCCTGGCCTGGGCGCTGGGGCCGCCGCGTGCGAATCTGGTGGCACCTTCGCGCCTGTCTCGCTGCTTTCGATAAGTCTCTAGCCATTTAAAATTTTTGATGACCTGCTGCGACGCTTTTTTTCTGGCAAGATAGTCTTGTAAATGCGGGCCAAGATCTGCACACTGGTATTTCGGTTTTTGGGGCCGCGGGCGGCGACGGGGCCCGTGCGTCCCAGCGCACATGTTCGGCGAGGCGGGGCCTGCGAGCGCGGCCACCGAGAATCGGACGGGGGTAGTCTCAAGCTGGCCGGCCTGCTCTGGTGCCTGGCCTCGCGCCGCCGTGTATCGCCCCGCCCTGGGCGGCAAGGCTGGCCCGGTCGGCACCAGTTGCGTGAGCGGAAAGATGGCCGCTTCCCGGCCCTGCTGCAGGGAGCTCAAAATGGAGGACGCGGCGCTCGGGAGAGCGGGCGGGTGAGTCACCCACACAAAGGAAAAGGGCCTTTCCGTCCTCAGCCGTCGCTTCATGTGACTCCACGGAGTACCGGGCGCCGTCCAGGCACCTCGATTAGTTCTCGAGCTTTTGGAGTACGTCGTCTTTAGGTTGGGGGGAGGGGTTTTATGCGATGGAGTTTCCCCACACTGAGTGGGTGGAGACTGAAGTTAGGCCAGCTTGGCACTTGATGTAATTCTCCTTGGAATTTGCCCTTTTTGAGTTTGGATCTTGGTTCATTCTCAAGCCTCAGACAGTGGTTCAAAGTTTTTTTCTTCCATTTCAGGTCCCGGGTAACTGATCATAATTCGACCCAAGTTTGTACAAAAAAGCAGGCTGATTACCGGAGAATTCCAATTGGCGGCCGCTAATACGACTCACTATAGGGAGAACCGGTGCCACCATGAAAGTGACCAAGGTCGACGGCATCAGCCACAAGAAGTACATCGAAGAGGGCAAGCTCGTGAAGTCCACCAGCGAGGAAAACCGGACCAGCGAGAGACTGAGCGAGCTGCTGAGCATCCGGCTGGACATCTACATCAAGAACCCCGACAACGCCTCCGAGGAAGAGAACCGGATCAGAAGAGAGAACCTGAAGAAGTTCTTTAGCAACAAGGTGCTGCACCTGAAGGACAGCGTGCTGTATCTGAAGAACCGGAAAGAAAAGAACGCCGTGCAGGACAAGAACTATAGCGAAGAGGACATCAGCGAGTACGACCTGAAAAACAAGAACAGCTTCTCCGTGCTGAAGAAGATCCTGCTGAACGAGGACGTGAACTCTGAGGAACTGGAAATCTTTCGGAAGGACGTGGAAGCCAAGCTGAACAAGATCAACAGCCTGAAGTACAGCTTCGAAGAGAACAAGGCCAACTACCAGAAGATCAACGAGAACAACGTGGAAAAAGTGGGCGGCAAGAGCAAGCGGAACATCATCTACGACTACTACAGAGAGAGCGCCAAGCGCAACGACTACATCAACAACGTGCAGGAAGCCTTCGACAAGCTGTATAAGAAAGAGGATATCGAGAAACTGTTTTTCCTGATCGAGAACAGCAAGAAGCACGAGAAGTACAAGATCCGCGAGTACTATCACAAGATCATCGGCCGGAAGAACGACAAAGAGAACTTCGCCAAGATTATCTACGAAGAGATCCAGAACGTGAACAACATCAAAGAGCTGATTGAGAAGATCCCCGACATGTCTGAGCTGAAGAAAAGCCAGGTGTTCTACAAGTACTACCTGGACAAAGAGGAACTGAACGACAAGAATATTAAGTACGCCTTCTGCCACTTCGTGGAAATCGAGATGTCCCAGCTGCTGAAAAACTACGTGTACAAGCGGCTGAGCAACATCAGCAACGATAAGATCAAGCGGATCTTCGAGTACCAGAATCTGAAAAAGCTGATCGAAAACAAACTGCTGAACAAGCTGGACACCTACGTGCGGAACTGCGGCAAGTACAACTACTATCTGCAAGTGGGCGAGATCGCCACCTCCGACTTTATCGCCCGGAACCGGCAGAACGAGGCCTTCCTGAGAAACATCATCGGCGTGTCCAGCGTGGCCTACTTCAGCCTGAGGAACATCCTGGAAACCGAGAACGAGAACGGTATCACCGGCCGGATGCGGGGCAAGACCGTGAAGAACAACAAGGGCGAAGAGAAATACGTGTCCGGCGAGGTGGACAAGATCTACAATGAGAACAAGCAGAACGAAGTGAAAGAAAATCTGAAGATGTTCTACAGCTACGACTTCAACATGGACAACAAGAACGAGATCGAGGACTTCTTCGCCAATATCGATGAGGCCATCTCTAGTATTGCTCACGGCATCGTGCATTTCAACCTGGAACTGGAAGGCAAGGACATCTTCGCCTTCAAGAATATCGCCCCCAGCGAGATCTCCAAGAAGATGTTTCAGAACGAAATCAACGAAAAGAAGCTGAAGCTGAAAATCTTCAAGCAGCTGAACAGCGCCAACGTGTTCAACTACTACGAGAAGGATGTGATCATCAAGTACCTGAAGAATACCAAGTTCAACTTCGTGAACAAAAACATCCCCTTCGTGCCCAGCTTCACCAAGCTGTACAACAAGATTGAGGACCTGCGGAATACCCTGAAGTTTTTTTGGAGCGTGCCCAAGGACAAAGAAGAGAAGGACGCCCAGATCTACCTGCTGAAGAATATCTACTACGGCGAGTTCCTGAACAAGTTCGTGAAAAACTCCAAGGTGTTCTTTAAGATCACCAATGAAGTGATCAAGATTAACAAGCAGCGGAACCAGAAAACCGGCCACTACAAGTATCAGAAGTTCGAGAACATCGAGAAAACCGTGCCCGTGGAATACCTGGCCATCATCCAGAGCAGAGAGATGATCAACAACCAGGACAAAGAGGAAAAGAATACCTACATCGACTTTATTCAGCAGATTTTCCTGAAGGGCTTCATCGACTACCTGAACAAGAACAATCTGAAGTATATCGAGAGCAACAACAACAATGACAACAACGACATCTTCTCCAAGATCAAGATCAAAAAGGATAACAAAGAGAAGTACGACAAGATCCTGAAGAACTATGAGAAGCACAATCGGAACAAAGAAATCCCTCACGAGATCAATGAGTTCGTGCGCGAGATCAAGCTGGGGAAGATTCTGAAGTACACCGAGAATCTGAACATGTTTTACCTGATCCTGAAGCTGCTGAACCACAAAGAGCTGACCAACCTGAAGGGCAGCCTGGAAAAGTACCAGTCCGCCAACAAAGAAGAAACCTTCAGCGACGAGTTGGAACTGATCAACCTGCTGAACCTGGACAACAACAGAGTGACCGAGGACTTCGAGCTGGAAGCCAACGAGATCGGCAAGTTCCTGGACTTCAACGAAAACAAAATCAAGGACCGGAAAGAGCTGAAAAAGTTCGACACCAACAAGATCTATTTCGACGGCGAGAACATCATCAAGCACCGGGCCTTCTACAATATCAAGAAATACGGCATGCTGAATCTGCTGGAAAAGATCGCCGATAAGGCCAAGTATAAGATCAGCCTGAAAGAACTGAAAGAGTACAGCAACAAGAAGAATGAGATTGAAAAGAACTACACCATGCAGCAGAACCTGCACCGGAAGTACGCCAGACCCAAGAAGGACGAAAAGTTCAACGACGAGGACTACAAAGAGTATGAGAAGGCCATCGGCAACATCCAGAAGTACACCCACCTGAAGAACAAGGTGGAATTCAATGAGCTGAACCTGCTGCAGGGCCTGCTGCTGAAGATCCTGCACCGGCTCGTGGGCTACACCAGCATCTGGGAGCGGGACCTGAGATTCCGGCTGAAGGGCGAGTTTCCCGAGAACCACTACATCGAGGAAATTTTCAATTTCGACAACTCCAAGAATGTGAAGTACAAAAGCGGCCAGATCGTGGAAAAGTATATCAACTTCTACAAAGAACTGTACAAGGACAATGTGGAAAAGCGGAGCATCTACTCCGACAAGAAAGTGAAGAAACTGAAGCAGGAGAAGAAAGACCTGTATATTGCAAACTACATCGCTCACTTTAATTATATTCCTCATGCCGAGATTAGCCTGCTGGAAGTGCTGGAAAACCTGCGGAAGCTGCTGTCCTACGACCGGAAGCTGAAGAACGCCATCATGAAGTCCATCGTGGACATTCTGAAAGAATACGGCTTCGTGGCCACCTTCAAGATCGGCGCTGACAAGAAGATCGAAATCCAGACCCTGGAATCAGAGAAGATCGTGCACCTGAAGAATCTGAAGAAAAAGAAACTGATGACCGACCGGAACAGCGAGGAACTGTGCGAACTCGTGAAAGTCATGTTCGAGTACAAGGCCCTGGAAACTAGTGGATCCGGACCTAAGAAAAAGAGGAAGGTGTAATCTAGATAGTGACTCGAGACTAGATAACTGATCTACCCAGCTTTCTTGTACAAAGTGGTACGCGTGAATTCACTCCTCAGGTGCAGGCTGCCTATCAGAAGGTGGTGGCTGGTGTGGCCAATGCCCTGGCTCACAAATACCACTGAGATCTTTTTCCCTCTGCCAAAAATTATGGGGACATCATGAAGCCCCTTGAGCATCTGACTTCTGGCTAATAAAGGAAATTTATTTTCATTGCAATAGTGTGTTGGAATTTTTTGTGTCTCTCACTCGGAAGGACATATGGGAGGGCAAATCATTTAAAACATCAGAATGAGTATTTGGTTTAGAGTTTGGCAACATATGCCCATATGCTGGCTGCCATGAACAAAGGTTGGCTATAAAGAGGTCATCAGTATATGAAACAGCCCCCTGCTGTCCATTCCTTATTCCATAGAAAAGCCTTGACTTGAGGTTAGATTTTTTTTATATTTTGTTTTGTGTTATTTTTTTCTTTAACATCCCTAAAATTTTCCTTACATGTTTTACTAGCCAGATTTTTCCTCCTCTCCTGACTACTCCCAGTCATAGCTGTCCCTCTTCTCTTATGGAGATCCCTCGACCTGCAGCCCAAGCTTGGCGTAATCATGGTCATAGCTGTTTCCTGTGTGAAATTGTTATCCGCTCACAATTCCACACAACATACGAGCCGGAAGCATAAAGTGTAAAGCCTGGGGTGCCTAATGAGTGAGCTAACTCACATTAATTGCGTTGCGCTCACTGCCCGCTTTCCAGTCGGGAAACCTGTCGTGCCAGCGGATCGACAGTACTAAGCTTGGTGCGTTTTTATGCTTGTAGTATTGTATAATGTTTTTAAGATCCTTAATTAATAGGGATAA*

**Supplementary Table.3 sgRNA sequences**

| NC sgRNA | TGACGCTTAACGACTGTTATGT |
| --- | --- |
| EGFP sgRNA | CGCCCTTGCTCACCATGGTGGC |
| Fluc sgRNA | TGGCTTTACCAACAGTACCGGAA |
| P53 sgRNA | ATCATCCATTGCTTGGGACGG |
| PTEN sgRNA | ATGGCTGTCATGTCTGGGAGC |

**Supplementary Table.4**

**The statistical tables presenting the docking scores (kcal/mol) and confidence levels of aptamers:**

| Aptamer | Docking Score | Confidence Score |
| --- | --- | --- |
| 1 | -320.00 | 0.9677 |
| 2 | -270.67 | 0.9178 |
| 3 | -267.36 | 0.9127 |
| 4 | -301.89 | 0.9543 |
| 5 | -247.22 | 0.8748 |

The docking scores were calculated using the HDOCK server and further refined using I-TASSER to predict aptamer binding to the dCas13a protein. The docking scores represent the binding free energy (kcal/mol), with more negative values indicating stronger predicted interactions. The confidence scores, also listed in the table, reflect the reliability of the predicted binding conformation based on model consistency and predicted structural quality. These docking results complement the SPR findings and are intended to support the predicted structural interaction models shown in Figure S2C. By combining computational docking with experimental validation, this table serves to substantiate the structural feasibility of using these aptamers for enhancing ETTAS functionality.

**Supplementary Figure 1. The detailed profile of vectors.**


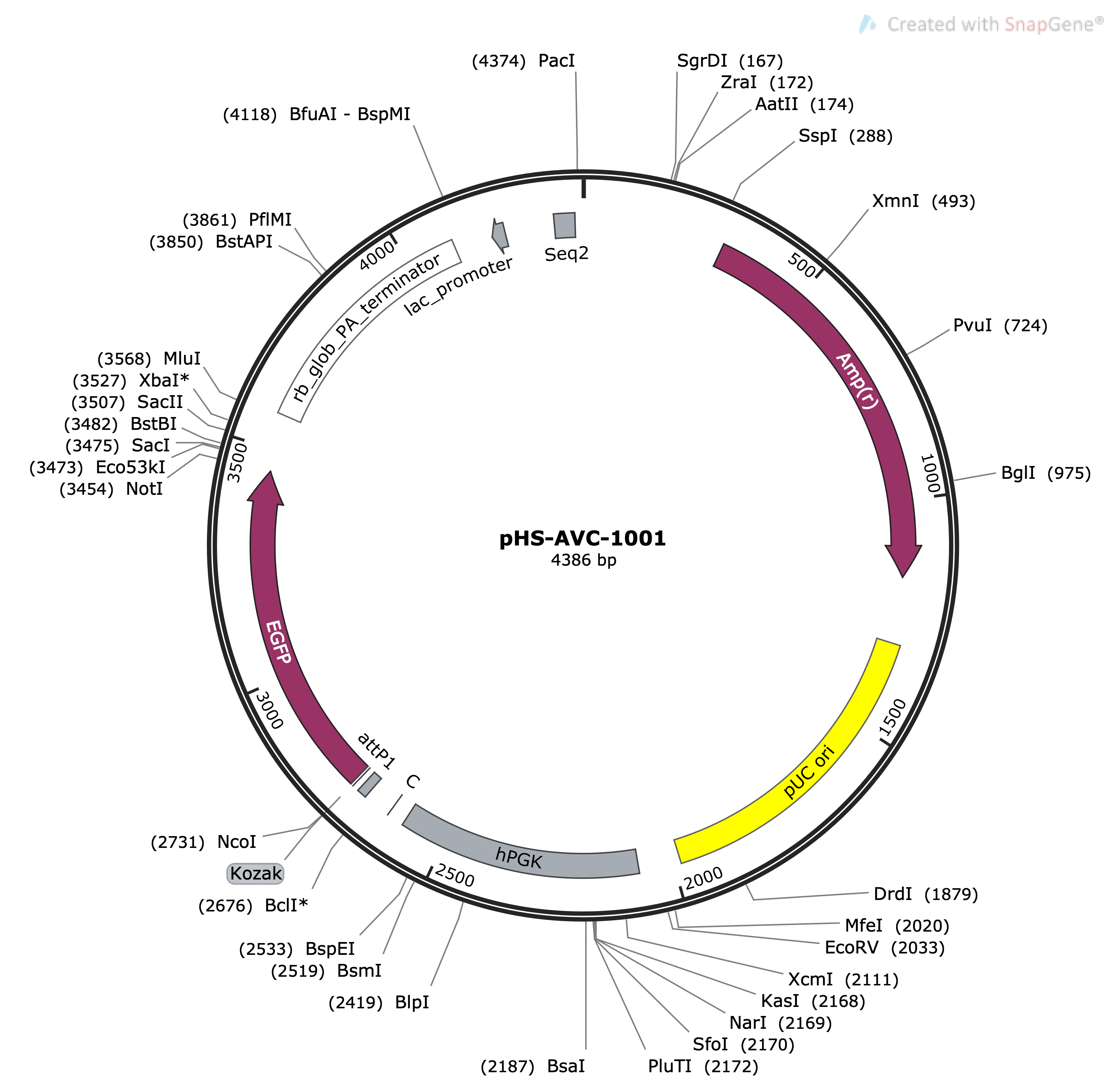

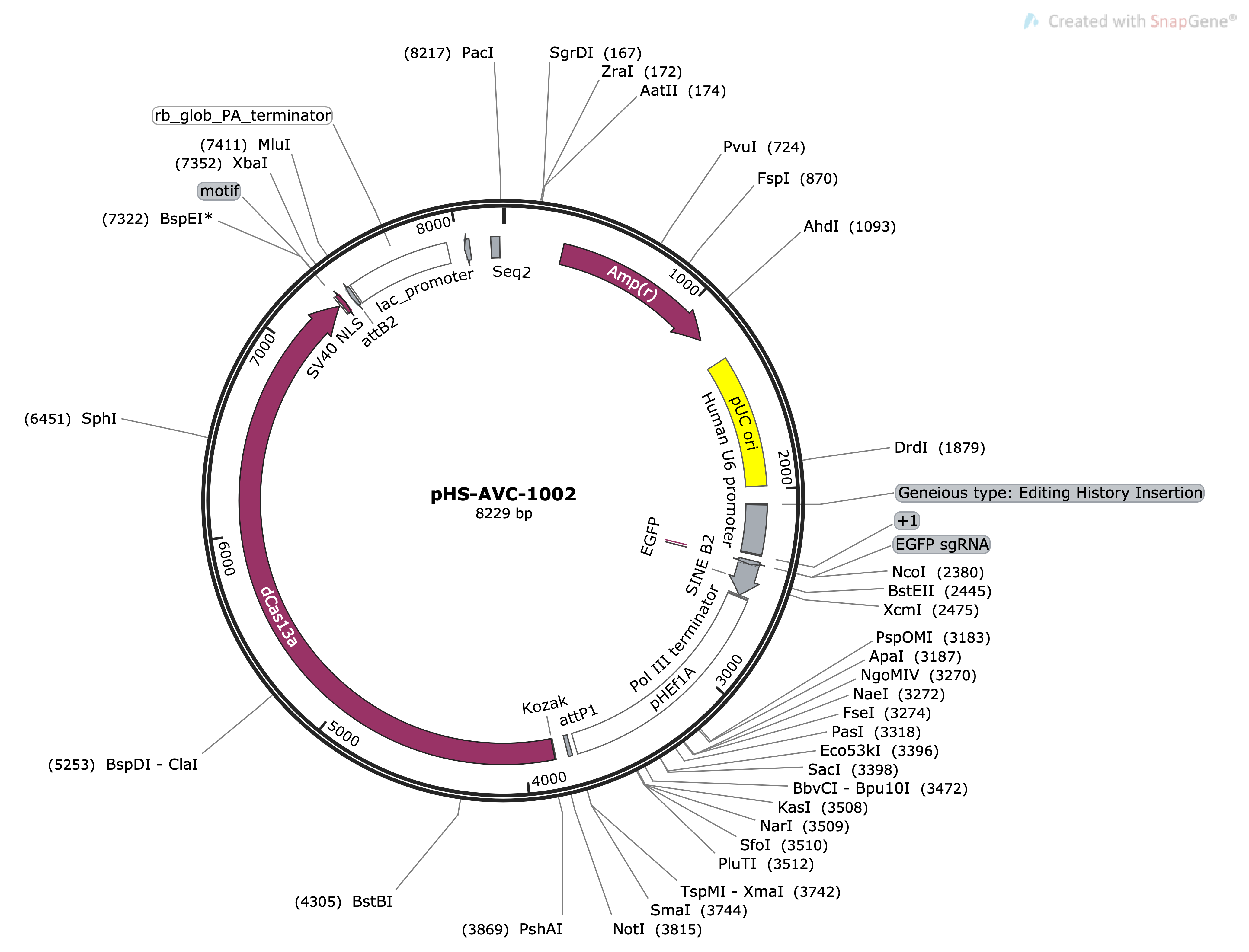


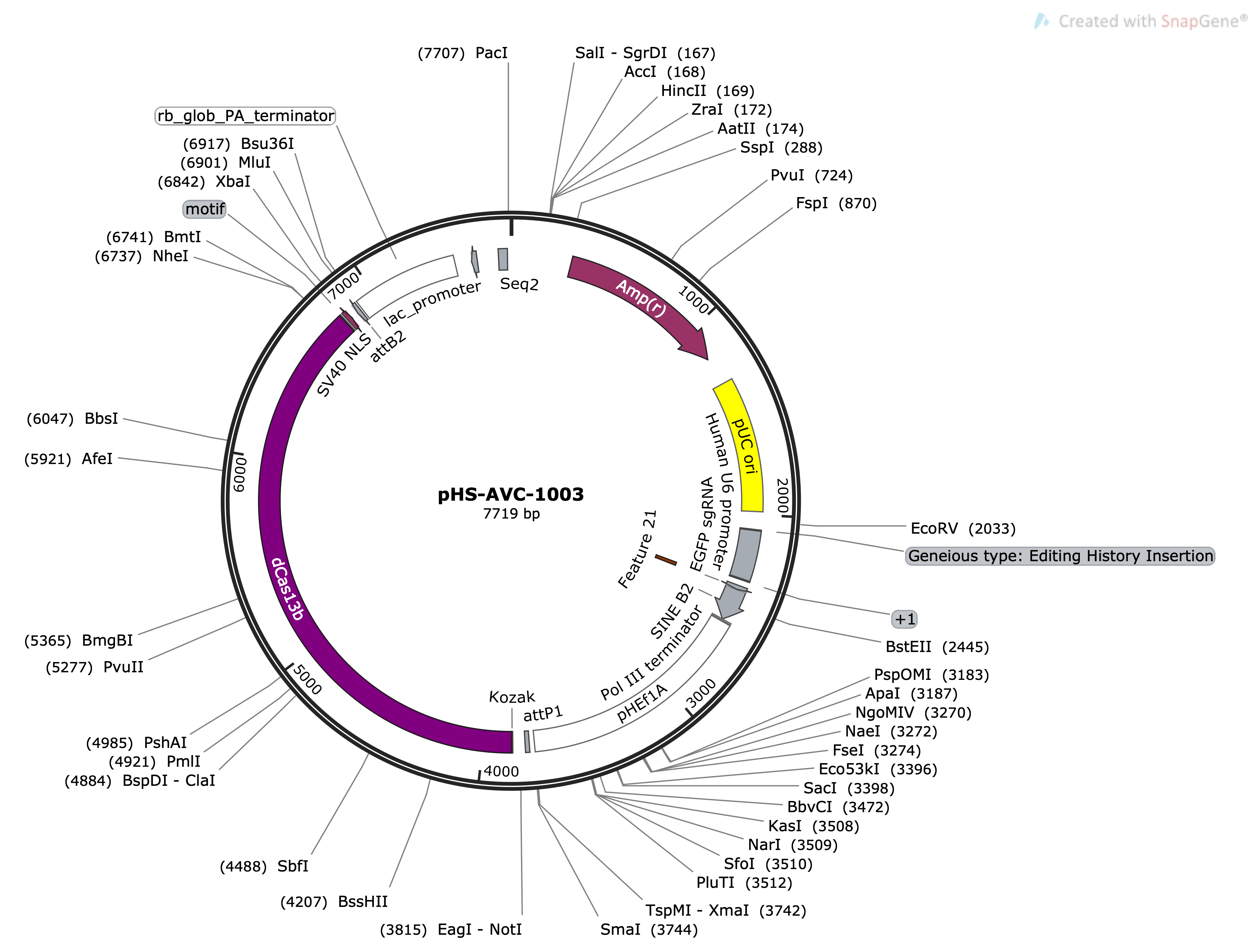

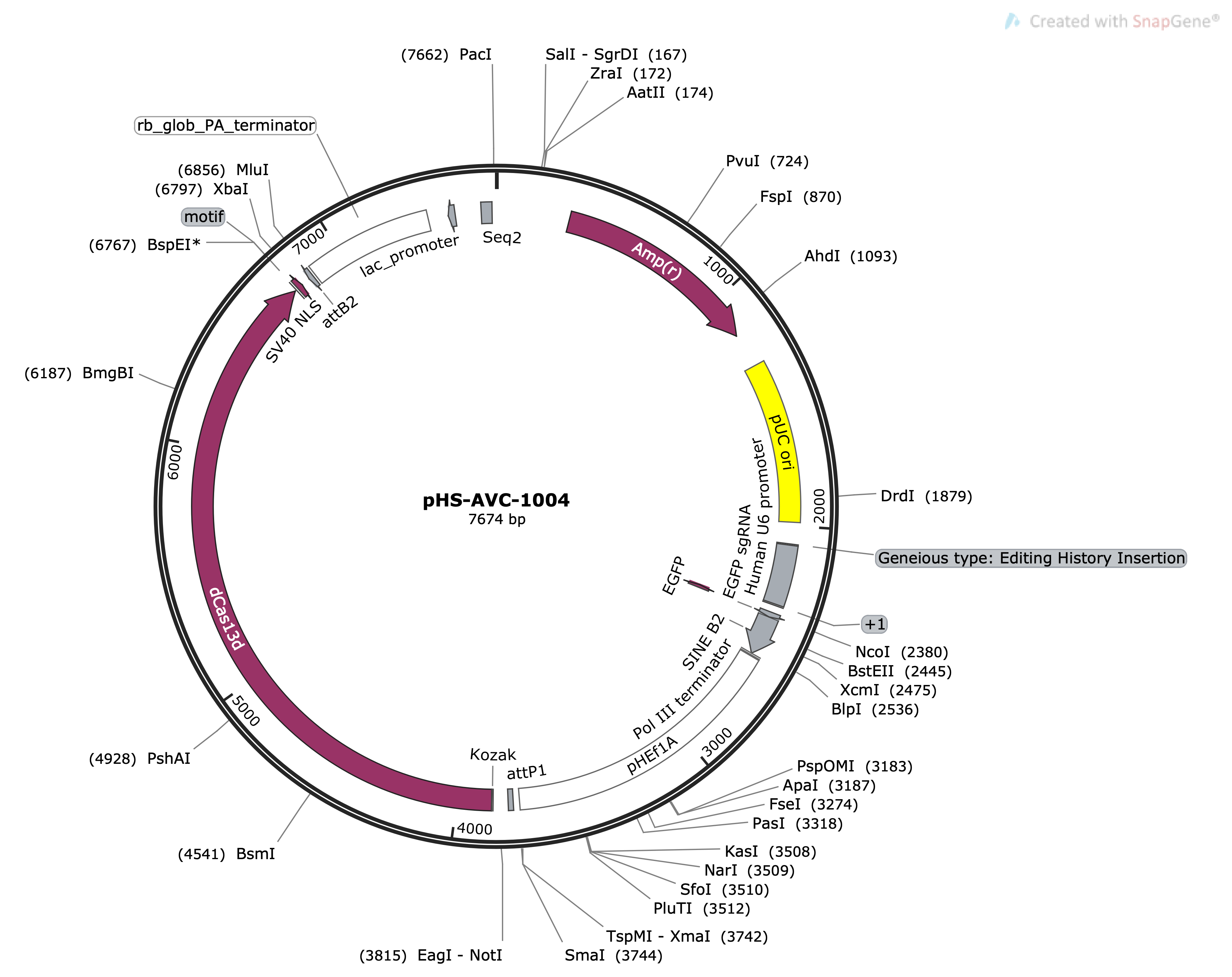


**Supplementary Figure 2. Tandem SINEB2 optimization and structural characterization of dCas13a-binding aptamers.**


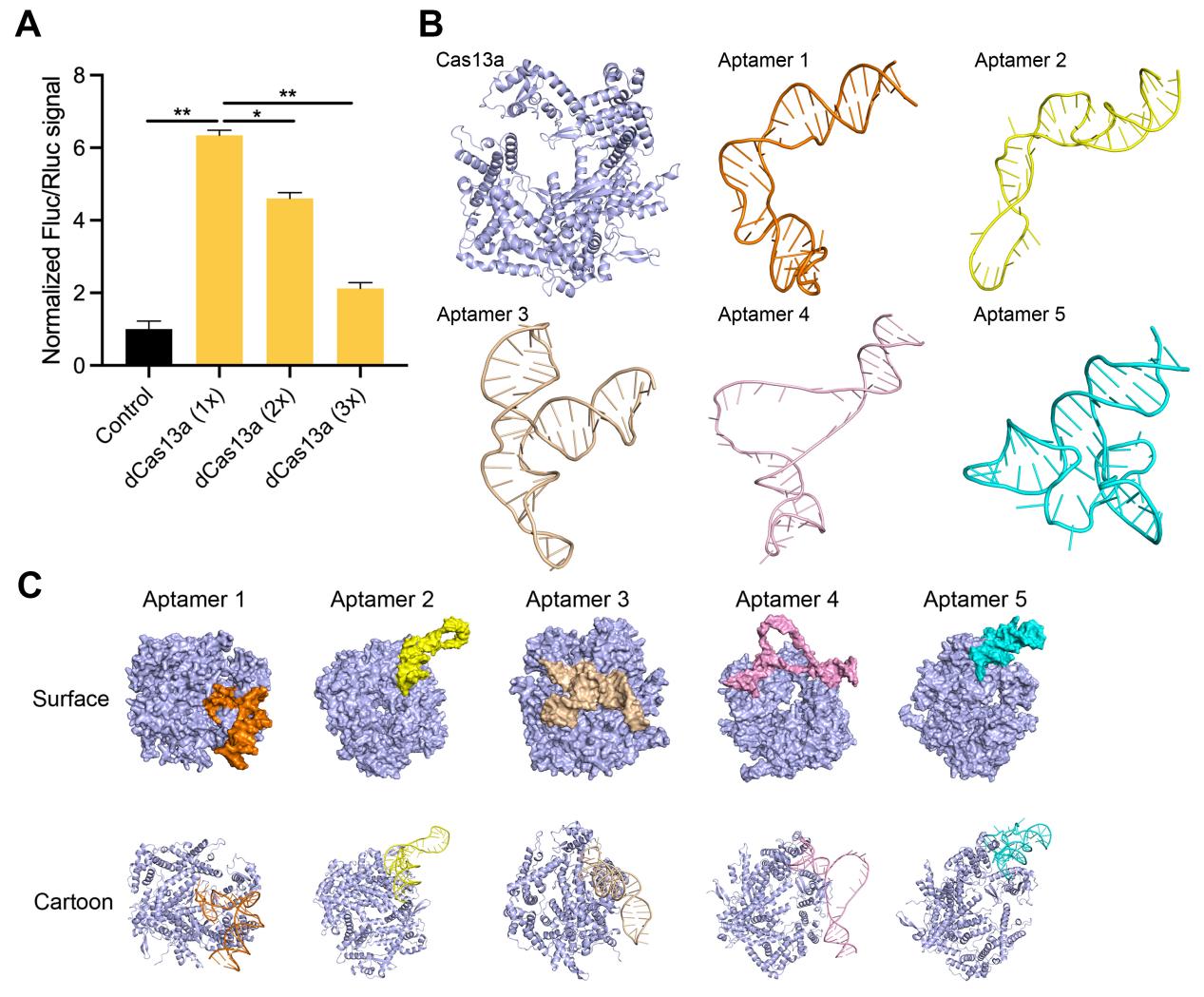


**(A)** Comparison of dCas13a-based translational activation constructs containing one, two, or three tandem SINEB2 elements using the Fluc/Rluc reporter assay. Increasing the number of tandem SINEB2 elements did not further enhance translational activation and instead reduced reporter activity. **(B)** Predicted RNA secondary structures of Aptamers 1-5 identified after DNA SELEX screening against dCas13a. **(C)** Representative docking models of Aptamers 1-5 with dCas13a shown in surface and cartoon representations.

**Supplementary Figure 3. Analysis of SPR measurement results.**

**A. Cas13a-aptamer 1 concentration gradient KD is 3.684 nM**

**
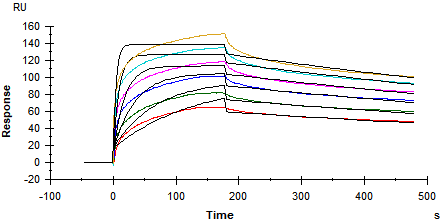

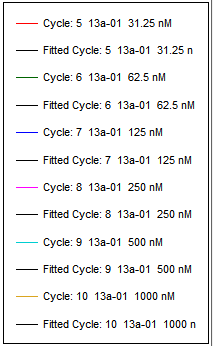
**

**
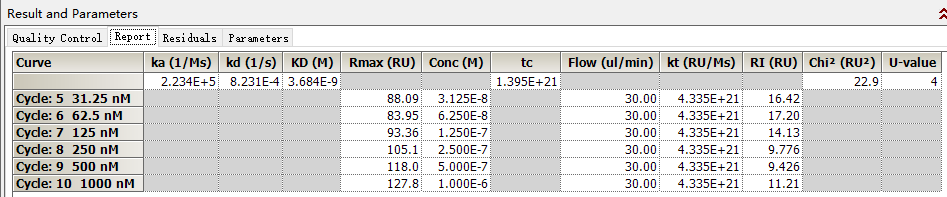
**

**B. Cas13a-aptamer 2 concentration gradient KD is 2.497 nM**

**
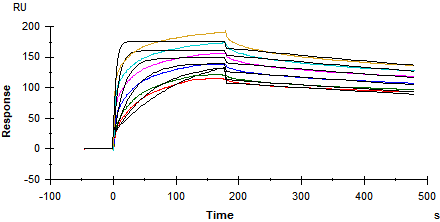

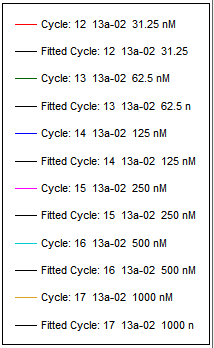
**

**
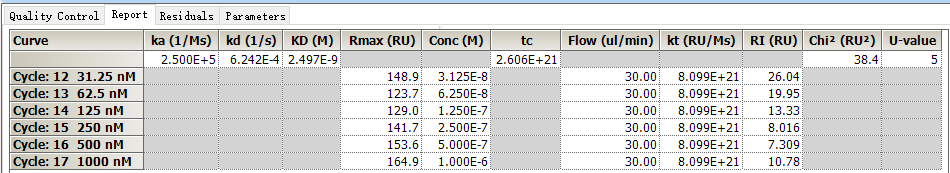
**

**C. Cas13a-aptamer 3 concentration gradient KD is 5.268 nM**

**
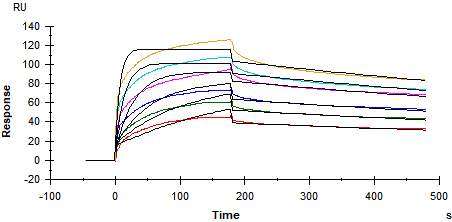

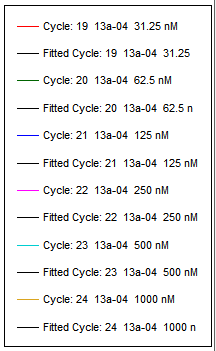
**

**
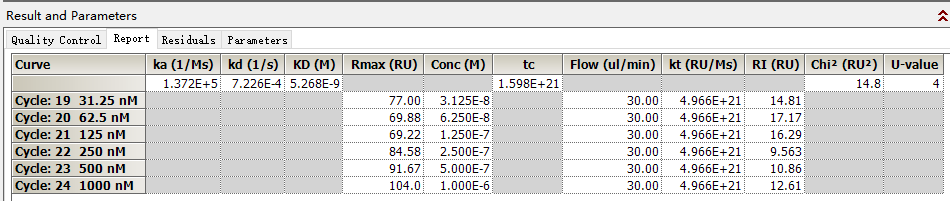
**

**D. Cas13a-aptamer 4 concentration gradient KD is 5.910 nM**

**
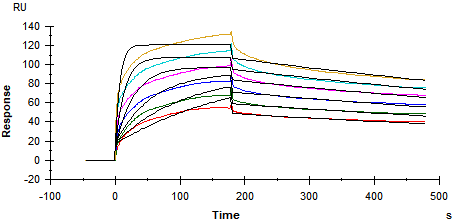

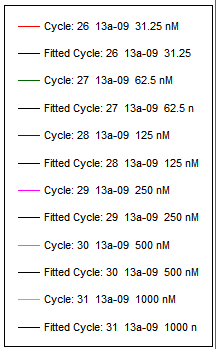
**

**
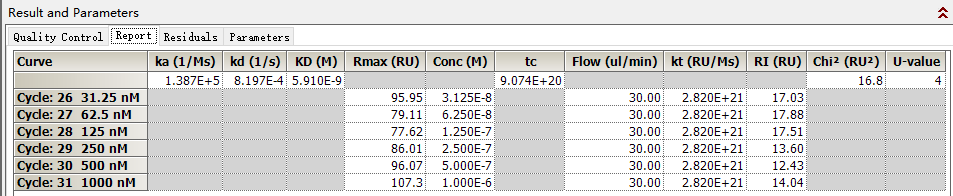
**

**E. Cas13a-aptamer 5 concentration gradient KD is 4.722 nM**


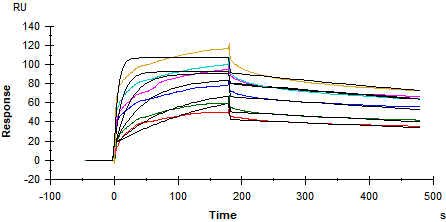

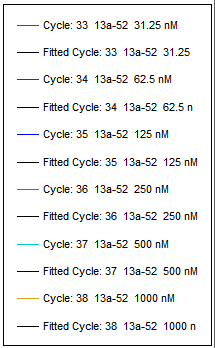


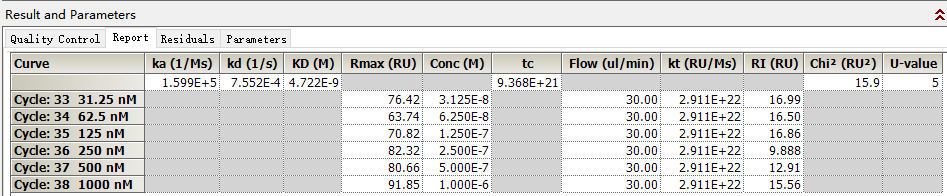


**Supplementary Figure 4. Predicted amino acid-nucleotide contact patterns between aptamers and the Cas13a protein based on molecular docking.**

**A. The interaction pattern of Cas13a protein with aptamer 1 molecule.**

**
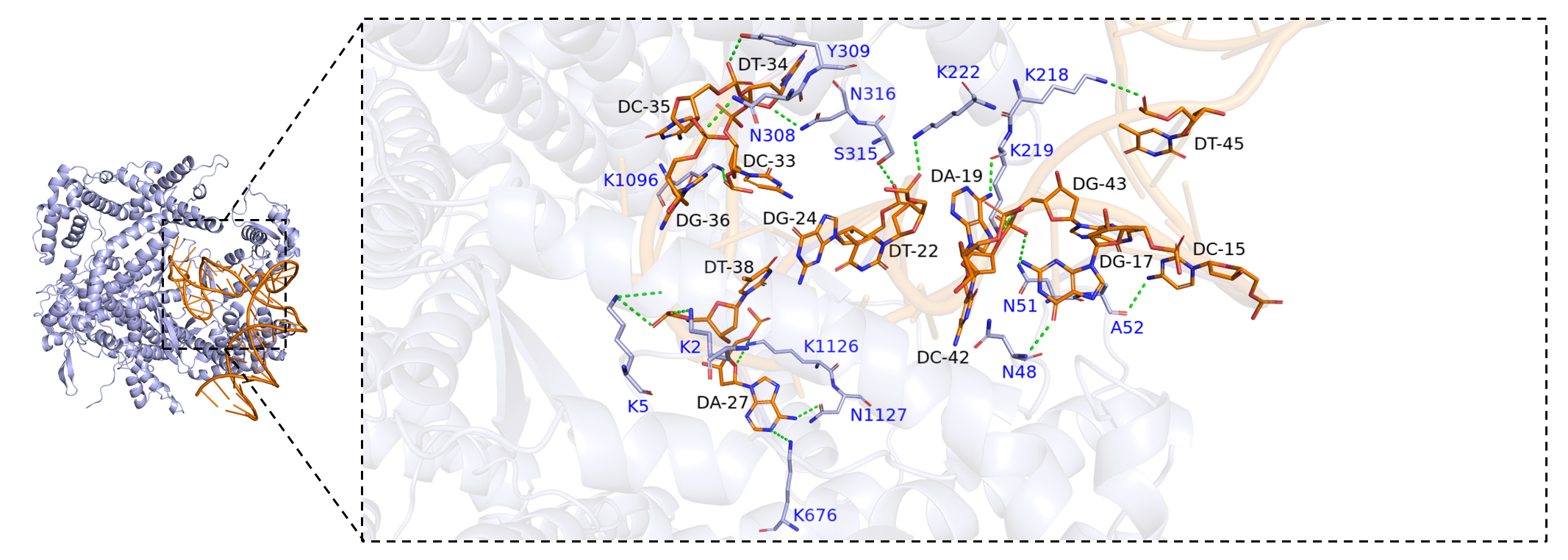
**

**B. The interaction pattern of Cas13a protein with aptamer 2 molecule.**

**
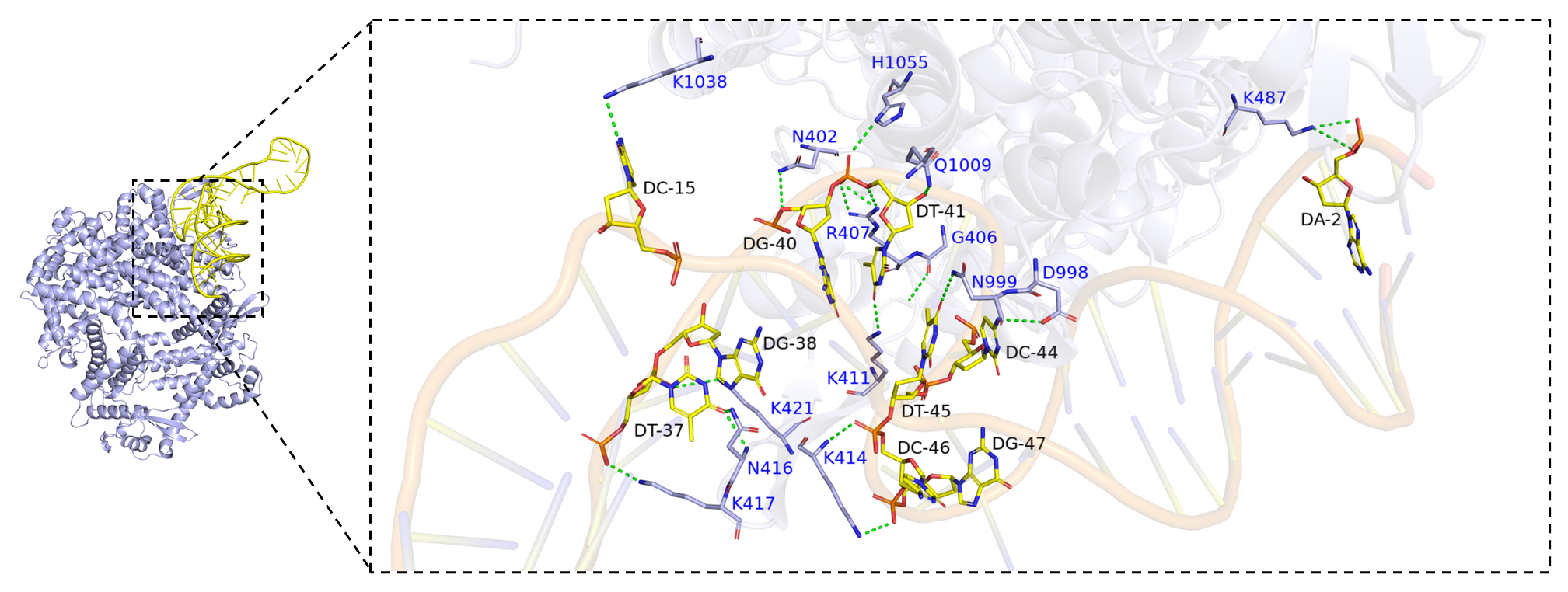
**

**C. The interaction pattern of Cas13a protein with aptamer 3 molecule.**

**
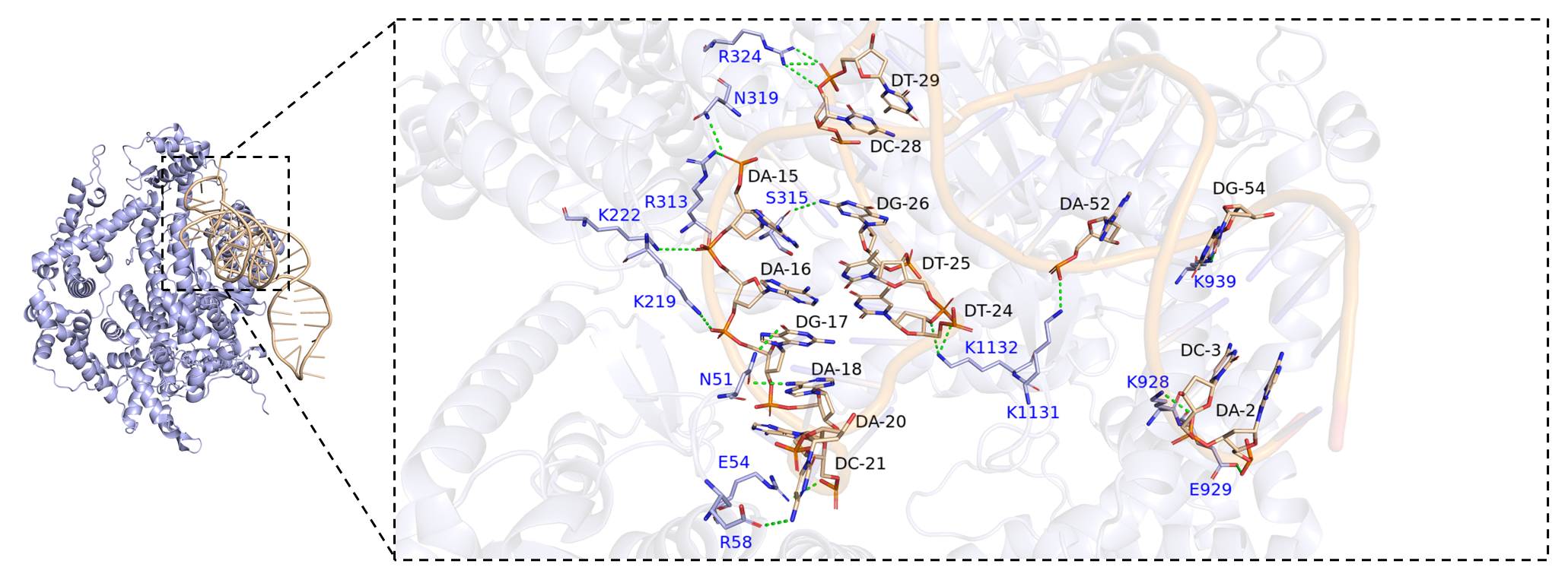
**

**D. The interaction pattern of Cas13a protein with aptamer 4 molecule.**

**
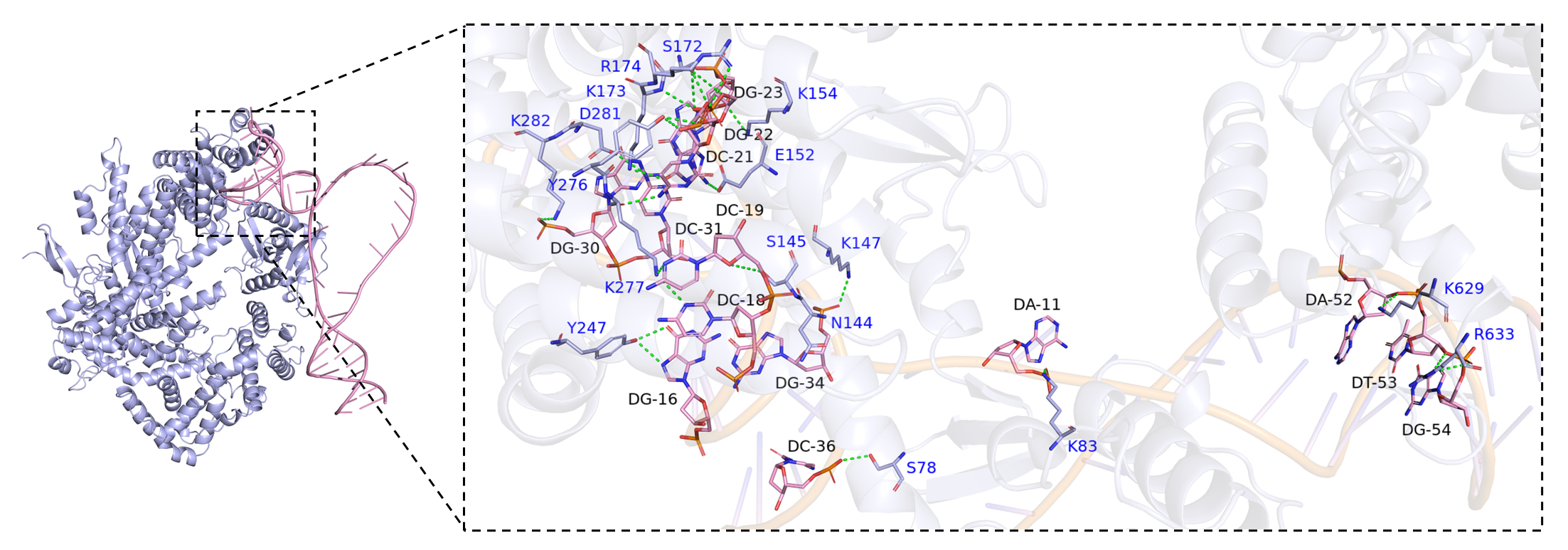
**

**E. The interaction pattern of Cas13a protein with aptamer 5 molecule.**

**
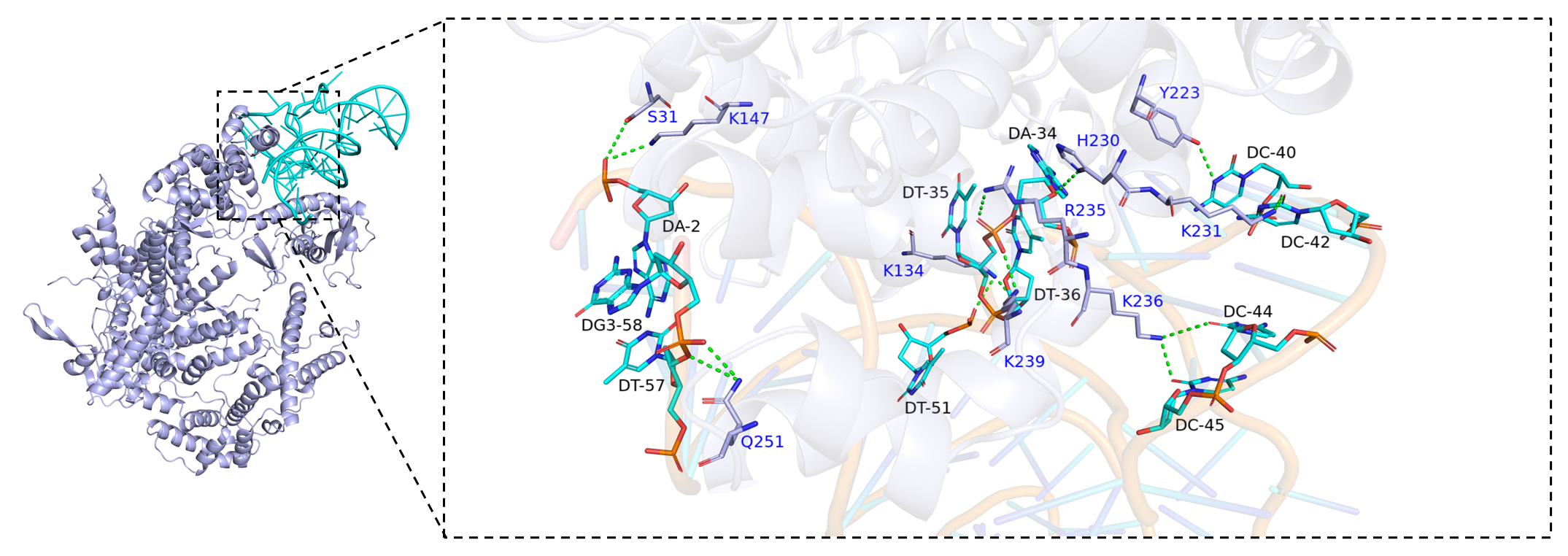
**

**Supplementary Figure 5. Cold-competitor bio-layer interferometry confirms the specificity of RNA-form Aptamer2 WT binding to dCas13a**


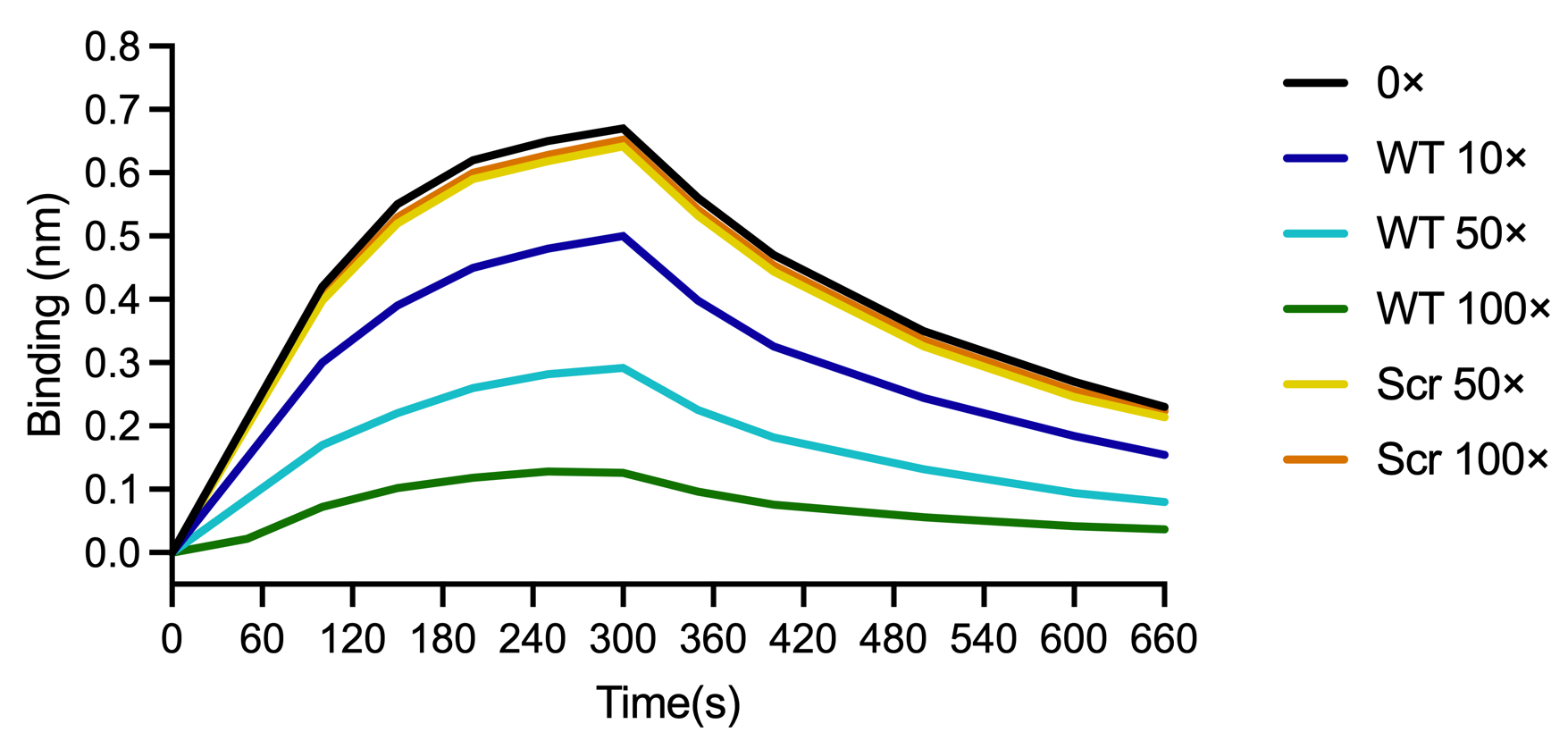


Bio-layer interferometry (BLI) sensorgrams showing binding of purified apo dCas13a to immobilized 5′-biotinylated RNA-form Aptamer2_WT in the presence of unlabeled cold competitors. Binding in the absence of competitor (0×) is shown for comparison. Increasing molar excesses (10×, 50×, and 100×) of unlabeled Aptamer2_WT progressively reduced the binding response in a dose-dependent manner, whereas excess scrambled RNA (50× and 100×) had only minimal effects. These results indicate that binding of dCas13a to RNA-form Aptamer2_WT is sequence-specific and can be specifically competed by unlabeled cognate aptamer.

**Supplementary Figure 6. High-throughput sequencing analysis of aptamer enrichment during SELEX.**
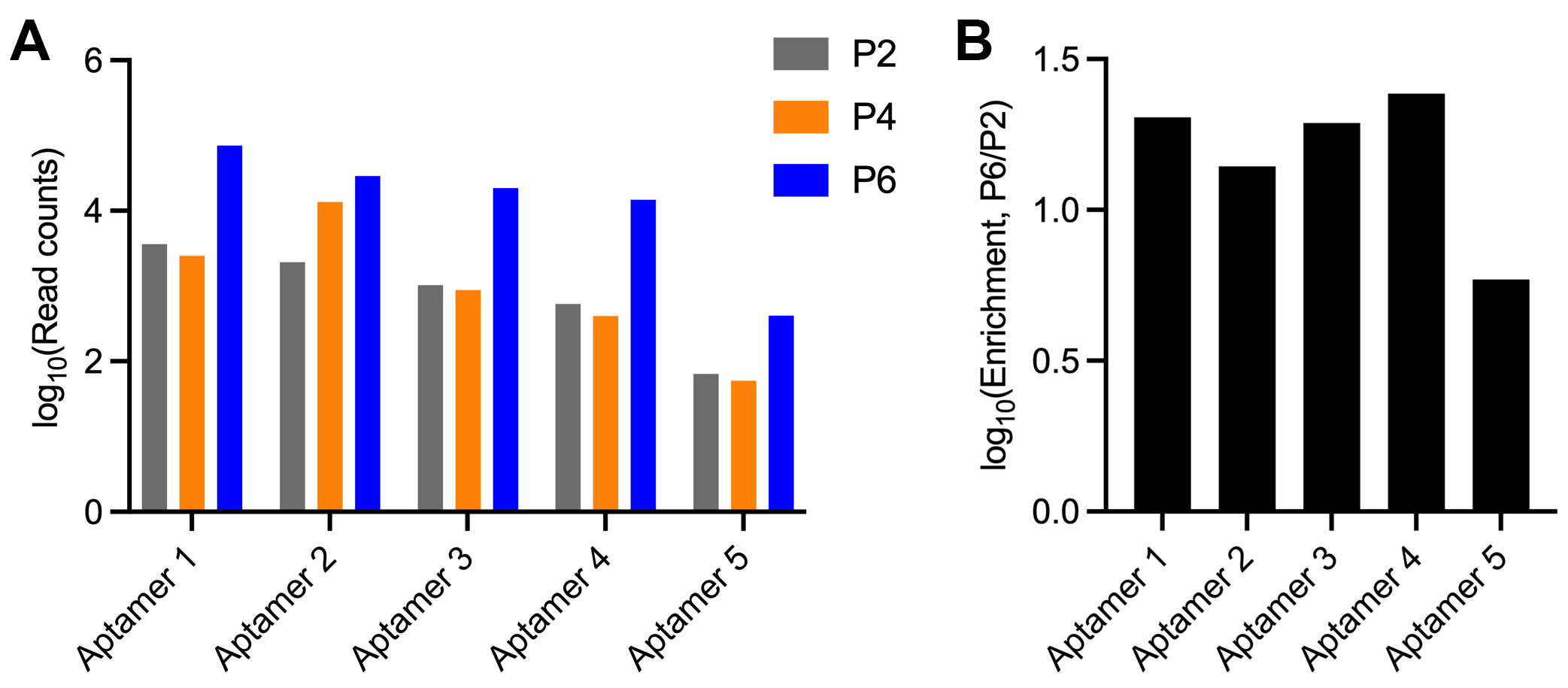


**(A)** Log10-transformed read counts of representative aptamer candidates across selection rounds (P2, P4, and P6), showing progressive enrichment of specific sequences. **(B)** Log10-transformed enrichment ratios (P6/P2) of the same aptamers, highlighting differential amplification efficiencies during selection.

**Supplementary Figure 7. Functional validation of ETTAS-mediated activation of endogenous P53 and downstream targets in bladder cancer cells.**


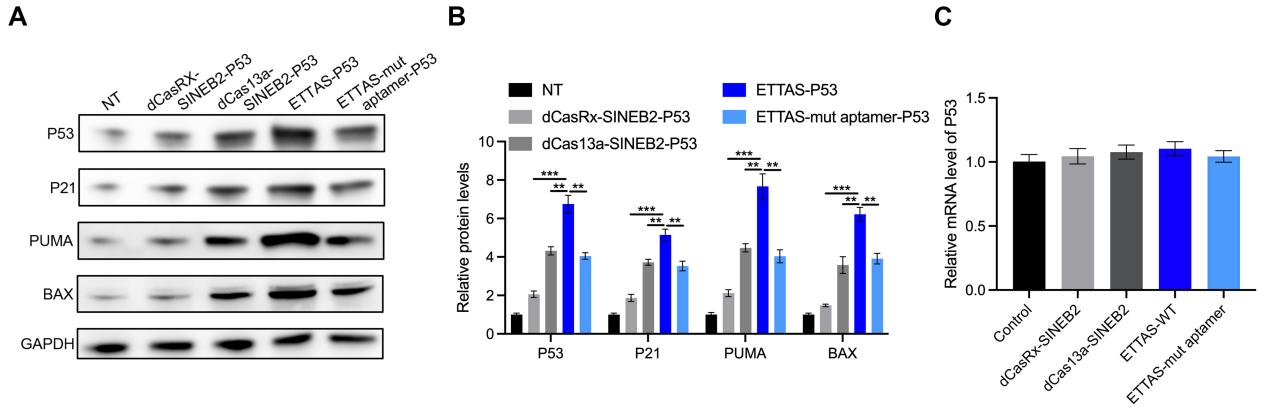
**(A)** Representative western blots showing protein levels of P53 and its downstream effectors P21, PUMA, and BAX in T24 bladder cancer cells transfected with non-targeting control (NT), dCasRx-SINEB2-P53, dCas13a-SINEB2-P53, ETTAS-P53, or ETTAS carrying a mutant Aptamer2 module. GAPDH serves as a loading control. **(B)** Quantification of protein expression from (A), normalized to GAPDH. ETTAS-P53 induces stronger upregulation of P53, P21, PUMA, and BAX compared with the indicated control constructs. **(C)** RT-qPCR analysis of relative P53 mRNA levels in the same experimental groups, showing no significant differences among groups. Data represent mean ± SD from three independent biological replicates, with two technical replicates per biological replicate. Statistical comparisons were performed using one-way ANOVA followed by Tukey’s multiple-comparisons test. Significance levels are indicated as **P < 0.01 and ***P < 0.001.
